# Risk and safety requirements for diagnostic and therapeutic procedures in allergology: World Allergy Organization Statement

**DOI:** 10.1186/s40413-016-0122-3

**Published:** 2016-10-12

**Authors:** Marek L. Kowalski, Ignacio Ansotegui, Werner Aberer, Mona Al-Ahmad, Mubeccel Akdis, Barbara K. Ballmer-Weber, Kirsten Beyer, Miguel Blanca, Simon Brown, Chaweewan Bunnag, Arnaldo Capriles Hulett, Mariana Castells, Hiok Hee Chng, Frederic De Blay, Motohiro Ebisawa, Stanley Fineman, David B. K. Golden, Tari Haahtela, Michael Kaliner, Connie Katelaris, Bee Wah Lee, Joanna Makowska, Ulrich Muller, Joaquim Mullol, John Oppenheimer, Hae-Sim Park, James Parkerson, Giovanni Passalacqua, Ruby Pawankar, Harald Renz, Franziska Rueff, Mario Sanchez-Borges, Joaquin Sastre, Glenis Scadding, Scott Sicherer, Pongsakorn Tantilipikorn, James Tracy, Vera van Kempen, Barbara Bohle, G Walter Canonica, Luis Caraballo, Maximiliano Gomez, Komei Ito, Erika Jensen-Jarolim, Mark Larche, Giovanni Melioli, Lars K. Poulsen, Rudolf Valenta, Torsten Zuberbier

**Affiliations:** 1 Department of Immunology, Rheumatology & Allergy, Medical University of Lodz, 251 Pomorska Str, 92-213 Lodz, Poland; 2 Department of Allergy and Immunology, Hospital Quiron Bizkaia, Bilbao, Spain; 3 Department of Dermatology, Medical University of Graz, Graz, Austria; 4 Microbiology Department, Faculty of Medicine, Kuwait University, Kuwait City, Kuwait; 5 Swiss institute of Allergy & Asthma research, Davos, Switzerland; 6 Allergy Unit, Dermatology Clinic, University Hospital Zürich, University Zürich, Zürich, Switzerland; 7 Kirsten Beyer, Charité Universitätsmedizin Berlin, Klinik für Pädiatrie m.S. Pneumologie und Immunologie, Berlin, Germany; 8 Hospital Reg. Univ. Carlos Haya, Allergy Serv, Malaga, Spain; 9 Royal Perth Hospital, Department of Emergency Medicine, Perth, WA Australia; 10 Department of Otolaryngology, Siriraj Hospital, Mahidol University, Bangkok, Thailand; 11 Allergology Unit, Hospital San Juan de Dios, Caracas, Venezuela; 12 Brigham & Women’s Hospital, Harvard Medical School, Boston, MA USA; 13 Department of Rheumatology, Allergy & Immunology, Tan Tock Seng Hospital, Singapore, Singapore; 14 Hôpitaux Universitaires de Strasbourg, Chest Diseases Department, Strasbourg, France; 15 Department of Allergy, Clinical Research Center for Allergology and Rheumatology, Sagamihara National Hospital, Sagamihara, Kanagawa Japan; 16 Emory University School of Medicine, Atlanta Allergy & Asthma, Atlanta, Georgia; 17 Johns Hopkins University, Baltimore, MD USA; 18 Helsinki University Central Hospital, Helsinki, Finland; 19 Institute for Asthma & Allergy, Bethesda, MD USA; 20 Westmead Medical Centre, Westmead, NSW Australia; 21 Department of Paediatrics, Yong Loo Lin School of Medicine, National University of Singapore, Singapore, Singapore; 22 CSK, Department of Allergy & Clinical Immunology, Lodz, Poland; 23 Spitalnetz, Bern, Switzerland; 24 Rhinology Unit & Smell Clinic, ENT Department, Hospital Clínic, Clinical & Experimental Respiratory Immunoallergy, IDIBAPS, and CIBERES, Barcelona, Spain; 25 UMDNJ – Rutgers Medical School, c/o Pulmonary and Allergy Associates, Summit, New Jersey, USA; 26 Department of Internal Medicine, Ajou University School of Medicine, Suwon, South Korea; 27 University of South Florida, Tampa, FL USA; 28 Allergy and Respiratory Diseases, IRCCS San Martino Hospital IST, University of Genoa, Genoa, Italy; 29 Department of Pediatrics, Nippon Medical School, Tokyo, Japan; 30 Universitatsklinikum GI & MR GmbH, Institut fur Laboratoriumsmedizin & Path, Standort Marburg, Marburg, Germany; 31 Klinikum der Ludwig-Maximilians-Universitat, Klinik & Poliklinik fur Dermatologie & Allergologie, Munchen, Germany; 32 Allergy and Clinical Immunology Department, Centro Medico-Docente La Trinidadad, Caracas, Venezuela; 33 Allergy Department, Fundacion Jimenez Diaz, Universidad Autonoma de Madrid, CIBER de Enfermedades Respiratorias (CIBERES), Institute Carlos III, Madrid, Spain; 34 RNTNE Hospital, London, UK; 35 Division of Allergy and Immunology, Department of Pediatrics, Jaffe Food Allergy Institute, Icahn School of Medicine at Mount Sinai, New York, NY USA; 36 University of Nebraska, Omaha, NE USA; 37 Institute for Prevention and Occupational Medicine, German Social Accident Insurance, Ruhr-University Bochum (IPA), Bochum, Germany; 38 Division of Experimental Allergology, Department of Pathophysiology, Allergy Research Center of Pathophysiology, Infectiology & Immunology, Medical University of Vienna, Vienna, Austria; 39 Allergy & Respiratory Disease Clinic, DIMI – Department Int Med, University of Genoa, IRCCS AOU, San Martino – IST, Genoa, Italy; 40 Immunology Department, Universidad De Cartagena, Cartagena, Colombia; 41 Hospital San Bernardo, Salta, Argentina; 42 Department of Allergy, Aichi Children’s Health and Medical Center, Aichi, Japan; 43 Messerli Research Institute, Medical University Vienna, University Vienna, Vienna, Austria; 44 Department of Biochemistry and Biomedical Sciences, McMaster University, Hamilton, Canada; 45 Istituto Giannina Gaslini, Genoa, Italy; 46 Gentofte University Hospital, Lab for Allergology, Allergy Clinic, Hellerup, Denmark; 47 Department of Pathophysiology, AKH, Wien, Austria; 48 Campus Charite Mitte, Klinik fur Dermatologie & Allergologie, Berlin, Germany

## Abstract

One of the major concerns in the practice of allergy is related to the safety of procedures for the diagnosis and treatment of allergic disease. Management (diagnosis and treatment) of hypersensitivity disorders involves often intentional exposure to potentially allergenic substances (during skin testing), deliberate induction in the office of allergic symptoms to offending compounds (provocation tests) or intentional application of potentially dangerous substances (allergy vaccine) to sensitized patients. These situations may be associated with a significant risk of unwanted, excessive or even dangerous reactions, which in many instances cannot be completely avoided. However, adverse reactions can be minimized or even avoided if a physician is fully aware of potential risk and is prepared to appropriately handle the situation.

Information on the risk of diagnostic and therapeutic procedures in allergic diseases has been accumulated in the medical literature for decades; however, except for allergen specific immunotherapy, it has never been presented in a systematic fashion. Up to now no single document addressed the risk of the most commonly used medical procedures in the allergy office nor attempted to present general requirements necessary to assure the safety of these procedures.

Following review of available literature a group of allergy experts within the World Allergy Organization (WAO), representing various continents and areas of allergy expertise, presents this report on risk associated with diagnostic and therapeutic procedures in allergology and proposes a consensus on safety requirements for performing procedures in allergy offices. Optimal safety measures including appropriate location, type and required time of supervision, availability of safety equipment, access to specialized emergency services, etc. for various procedures have been recommended.

This document should be useful for allergists with already established practices and experience as well as to other specialists taking care of patients with allergies.

## Introduction

Over the last several decades allergy practice has been expanding worldwide in parallel with the increasing number of patients suffering from allergic diseases. It has been widely accepted that appropriate training and certification are necessary for the physician to correctly diagnose and manage allergic diseases. However, in some countries the allergy specialty is still poorly developed or does not exist. Allergy practice, therefore, varies from country to country and according to local regulations or traditions both trained allergists or physicians with other specialties are performing allergy procedures such as skin testing or immunotherapy. Furthermore, in several regions of the world the increasing number of allergy sufferers has not been matched by an appropriate supply of trained specialists; as a result, physicians without training in allergy including general practitioners and pediatricians will be assisting allergic patients more and more.

Doctors dealing with allergic diseases (certified allergists or other specialists trained in allergy) are employing, in the office, various diagnostic and therapeutic procedures which are associated with a significant risk of unwanted reactions for a patient. The reactions, depending on the procedure, may vary from local discomfort to severe anaphylaxis and death. Most unwanted reactions can be either avoided or the risk/symptom intensity may be minimized if the procedures are performed in an appropriate manner. However, certain risk of unwanted and or excessive reaction remains even if all precautions are respected. During some diagnostic procedures, called provocations (e.g. oral drug or food challenges), the allergist deliberately aims at inducing adverse symptoms which are mimicking those occurring at natural exposure and sometimes may be associated with a significant discomfort and even with some risk to the patient. In such situations unpleasant or even potentially dangerous symptoms are inherent to the procedure and cannot be completely avoided. Thus, it is critical that well established inclusion/exclusion criteria for the challenge are considered and the protocols of provocations are strictly followed. Furthermore the patient should be appropriately monitored by trained and experienced medical staff not only during the whole procedure but also for an appropriate time after its completion. Such precautions usually allow for a significant reduction of risk of unwanted or excessive symptoms.

The literature on the risk of allergic procedures exists, but it has not been recently reviewed in a systematic way. Furthermore, there is no available consensus on safety requirements for performing specific diagnostic procedures. Thus it is important to reach the consensus on optimal safety measures (e.g. appropriate location, type and required time of supervision, availability of safety equipment, access to specialized emergency services, etc.) for various procedures.

An international group of experts collaborating within the World Allergy Organization (WAO) presents this consensus report assessing risk and proposing safety requirements for performing procedures in allergy offices. This document refers to available literature and also to other documents and resources (e.g. local regulations) available to experts. Since for the majority of reviewed procedures no formal recommendations were available, the experts had to reach the consensus with regard to proposed recommendations. As a result, optimal safety measures for various procedures have been proposed.

This consensus, which is based on the recommendations of international experts, provides useful information for allergy specialists and all doctors who diagnoses and treat allergy patients. Moreover, the consensus has value for general allergy practice worldwide; thus WAO is an appropriate organization to provide it.

The following grading of recommendations on the safety measures has been presented based on the consensus reached by the expert panel:

A. Mandatory
B. Recommended
C. Suggested
D. non-required

Recommendations reported in the sections below have been summarized in Table 1 and 2.

**Table 1:** Summary of Safety Recommendations for Diagnostic Allergy Procedures *(for details, see the text)*

Section subtitle	Recommended site	Emergency equipment availability	Emergency staff (ICU) availability	Duration of supervised follow-up in the office after procedure	Comments
Skin testing (SPT and IDST)					
With Inhalant and food allergens	Both O and H	(a) mandatory	(c) not required	20 min	Field skin testing (prick test only), for epidemiology studies, may also be carried out by trained medical personnel.
Skin testing with hymenoptera venoms	Both O and H	(a) mandatory	(c) not required	20 min	
Skin testing with drugs	O or H depending on the risk assessment	(a) mandatory Comment: not applicable for patch testing	available on site (a) or available within 30 min (b) depending on the risk assessment	20 min	Patients at risk: Patients who are tested for anaphylactic reactions, or with a history of complicating conditions such as asthma, mastocytosis and severe cardiac disease
Skin testing with occupational allergens	Both O and H	(a) mandatory	(c) not required	20 min	
Skin testing with latex	Both O and H	(a) mandatory	(b) available within 30 min	20 min	For some patients waiting time should be extended to 40 min
Bronchial challenge with allergen	Both O and H^a^	(a) mandatory	(b) available within 30 min	7 h	^a^although outpatient clinic is acceptable the hospital setting is recommended
Bronchial challenge with lysine aspirin (Lys ASA BPT)	Both O and H	(a) mandatory	(b) available within 30 min	1 h	
Nonspecific bronchial provocation tests (NS-BPT)	Both O and H	(a) mandatory	(c) not required	not required	
Nasal allergen provocation tests	Both O and H	(a) mandatory	(b) available within 30 min	30 min	
Nasal aspirin provocation tests	Both O and H	(a) mandatory	(b) available within 30 min	30 min	
Nasal endoscopy	Both O and H	(d) not required	(c) not required	not required unless complications occur	
Food challenges	Both O and H	(a) mandatory	(a) available on site or (b) available within 30 min, depending on risk assessment	1-2 h after negative and 4 h after positive food challenge	
Oral drug provocation test	O or H depending on risk assessment	(a) mandatory	(a) available within 5 min, or (b) available within 30 min, depending on risk assessment	at least 2 h; hospitalization is recommended after severe reaction	
Insect sting challenge	Both O and H	(a) mandatory	(b) available within 30 min	at least 2 h until symptoms have disappeared	

O: Outpatient clinic

(a) mandatory

(b) recommended

(c) suggested

(d) not required

H: Hospital setting

(a) available on site (in less than 5 min)

(b) available within 30 min

(c) not required

**Table 2:** Summary of Safety Recommendations for Therapeutic Allergy Procedures *(for details, see the text)*

Section subtitle	Recommended site	Emergency equipment availability	Emergency Staff (ICU) Availability	Duration of supervised follow-up in the office after procedure	Comments
Subcutaneous immunotherapy with inhalant allergens	Both O and H	(a) mandatory	(a) available on site (in less than 5 min), or (b) available within 30 min, depending on risk assessment and the immunotherapy protocol used	30 min	
Venom immunotherapy	Both O and H	(a) mandatory	(b) should be available on site, (in less than 5 min) or within 30 min, depending on the risk assessment and the immunotherapy protocol used	30 min	
Drug desensitization procedures	O or H depending on risk assessment	(a) mandatory	(a) available on site (in less than 5 min)	30 min after acute reactions	Waiting time can be extended to 24-98 h for delayed reactions
Oral immunotherapy for food allergy	Both O and H	(a) mandatory	available within 5 minutess (a) or available within 30 min (b) depending on risk assessment	2 h	Currently not recommended for routine clinical use
Treatment with Anti-IgE and other biologicals		(a) mandatory	(b) available within 30 min	2 h for the first administration;30 min for succesive administration	
Treatment with products from human plasma		(a) mandatory	(b) available within 30 min	30 min	

O: Outpatient clinic

(a) mandatory

(b) recommended

(c) suggested

(d) not required

H: Hospital setting

(a) available on site (in less than 5 min)

(b) available within 30 min

(c) not required

## Diagnostic procedures

### Skin testing for inhalant and food allergens – Skin Prick Test (SPT) and Intradermal Skin Test (IDST)

#### Skin testing with inhalant and food allergens

##### Definition and short technical description

These in vivo tests are used for the detection of allergen specific IgE on the skin mast cells and confirmation of sensitization to a specific allergen [1].

*Skin Prick Test (SPT)*

Skin prick testing relies on the introduction of a very small amount of allergen extract into the epidermis using a disposable fine needle or lancet device, which is changed with each test allergen [2]. Besides metal devices, there are other varieties of commercially available skin prick devices. The incorporation of these devices into the protocol may require prior evaluation [3]. Skin prick should be applied carefully, as insufficient prick may produce false negative results, and induction of bleeding (too deep) may produce false positive results and bear the risk of systemic reactions. Allergens should be placed at least 2 cm apart to avoid overlapping responses between allergens tested. The results of skin prick testing are read at 15 min and the diameter of the resulting weal is recorded in two dimensions (longest and its orthogonal diameter). By convention, a positive test is one in which the mean of the two weal diameters is at least 3 mm greater than the negative control (saline) [4]. Positive and negative controls are critical to enable interpretation of test results. Ideally, the histamine control is read at 10 min. The test is usually performed on the volar aspect of the forearm but it is also performed on the back, especially in young children. There is a gradient of response when using the back – with larger responses in the lower third compared to the upper third [5]. Interpretation of results should consider the following factors: the allergen extracts used (standardized when available), the type of lancet device, the skin site chosen for testing, the clinical state of the patient and the medications used by the patient.

*Intradermal allergy testing (IDST)*

Intradermal allergy testing is a procedure where a small amount of diluted allergen is injected into the dermis. It increases the sensitivity but decreases the specificity of the test and is carried out with allergen concentrations 100 to 1000 times less than that used for skin prick tests. It has no place in aeroallergen (other than for research) and food allergen testing. It is most commonly used in testing for drug and venom allergy.

##### Clinical indications

SPTs may be used for the evaluation of allergen-specific IgE to inhalants, foods, drugs and venom in the following conditions: respiratory/inhalant allergy, food allergy, venom allergy, drug allergy.

IDSTs have a very high non-specific reaction rate and are not recommended for testing with inhalants or foods [6] and food allergens [7]. Moreover, intradermal tests carry a higher risk of adverse reactions than SPTs.

##### Age limitation

Testing can be performed from infants to the elderly. Infants and the elderly have smaller SPT weal responses, and prominent flare responses [8].

##### Description of adverse/unintended reactions associated with the procedure

SPT is considered a safe procedure, with minimal discomfort. Adverse events can occur but rarely. These are classified as allergic or non-allergic.

1. Type and spectrum of unintended allergic reactions
  Local:
    In some patients with marked sensitivity late phase local skin swelling (the IgE late phase response) consisting of tender and painful swelling may occur (seen more commonly with intradermal testing). Rarely, it could cause quite marked swelling and discomfort, but does not usually last more than 36 h [9].
  Systemic:
    Systemic reactions associated with SPTs, usually starting within 15 to 30 min, have been reported as case reports [10], in surveys and in prospective studies. Although systemic reactions may occur in any individual undergoing skin testing (both adults and children), specific risk factors should be taken into consideration when performing these tests (see section III).
  Fatal Reactions:
    Few fatal reactions as a result of skin testing have been described in the literature [11, 12]. Based on two large retrospective surveys by the American Academy of Allergy and Immunology in the US, seven fatalities have been described involving older children and adults. Six of these deaths involved intradermal testing to inhalants and food and one death involving skin prick testing performed with 90 allergens.
2. Type and spectrum of non-allergic reactions.
  These may include syncope (vasovagal syncope) and headache, Based on a prospective study in children [13] and a retrospective survey [11], all reported systemic and vasovagal syncope reactions related to skin testing occurred within 15 to 30 min of the test.
3. Prevalence and risk associated with the procedure.
  The prevalence of systemic reactions related to skin prick testing with inhalant and food allergens is low but not absent. It was estimated to be less than 0,055 % [14, 15]*.* The rate of systemic reactions requiring epinephrine was reported as 20 per 100,000 SPT visits [16]. The prevalence in young children appears to be higher with a reported rate of systemic reactions of 0.12 % [13] and 6.5 % in infants less than 6 months of age [17].
4. Risk factors for adverse/unintended reactions
  Systemic Reactions: [7, 9, 11, 12, 17]
  Infants especially <6 months
  Multiple allergens
  Previous history of anaphylaxis to food when testing for incriminating food
  Testing with fresh food (non-commercial extracts)
  Testing with non-standardized latex extracts
  Extensive eczema
  Uncontrolled asthma
  Intradermal Testing
  Vasovagal syncope [13]:
  Female sex
  Testing with multiple allergens

##### Institutional/organizational safety recommendations

Several guidelines for performing skin tests have been published:

- Allergy diagnostic testing: an updated practice parameter (Bernstein et al, 2008) [18]
  - Skin Prick Testing for the diagnosis of allergic diseases – A manual for practitioners (Australasian Society of Clinical Immunology and Allergy, 2013) [9]
  - Allergic Rhinitis and its Impact on Asthma; Practical guide to skin prick tests in allergy to aeroallergens. Allergy 2012;67:18-24 [19].

##### WAO safety recommendations

These recommendations are based on the rare occurrence of severe systemic reactions reported in retrospective surveys, one prospective study, and several case reports. Quality of evidence is high regarding the rare occurrence of systemic life threatening and fatal (1 case in the literature with skin prick test without intradermal test) reactions justify the need for facilities offering skin prick testing to have the following prerequisites for safety. There are no recommendations for intradermal testing as they are not indicated for inhalant and food testing [20].

1. Site:
  Both a hospital and outpatient clinic setting
  Field (skin prick test only), e.g. epidemiology studies, may also be carried out by trained medical personnel.
2. Personnel:
  Can be performed by trained nurse/technician under supervision of experienced physician
3. Emergency equipment availability:
  Should be available on site (mandatory)
4. Emergency staff (ICU) availability:
  Not required
5. Pretreatment:
  Not applicable
6. Duration of supervised follow up after the procedure:
  It is recommended that patients who have undergone skin prick testing and have positive results, who have asthma or a history of anaphylaxis, should remain in the centre for at least 20 min following completion of the skin prick test [9].
7. Contra-indications:
  Contraindications to skin prick testing may be categorized into:
  Clinical situations which interfere with the procedure or its interpretation. These include absence of normal skin, including dermatographism, use of medication that might inhibit skin prick responses.
  Relative contraindications related to safety/high risk situations. These include severe or unstable asthma and patients on beta-blockers. If SPT is considered to carry a significant risk e.g. in a highly sensitized patient or in a woman with unstable asthma in pregnancy then avoid performing the test.
8. Other considerations:
  In patients with a history of anaphylaxis, skin prick tests should be initiated with several serial 10-fold dilutions of the usual test concentration.

#### Skin testing with hymenoptera venoms

##### Definition and short technical description

Immediate hypersensitivity skin testing is performed with standard techniques and standard reporting of results. Venom skin testing may begin with prick/puncture tests using a venom concentration of 1,0- 100 mcg/ml, or with intradermal tests using venom concentration of 0.001 -1 mcg/ml. If the puncture test is negative, it is followed by an intradermal test using venom concentration of 0.01 mcg/ml. If the intradermal test using venom concentration of 0.01 mcg/ml is negative, it is repeated using concentrations of 0.1 mcg/ml, and then if necessary 1.0 mcg/ml. A positive puncture test has a wheal diameter at least 3 mm larger than the diluent (negative) control. Intradermal tests should introduce sufficient volume to give a 3–4 mm bleb (usually 0.02–0.03 ml). A positive intradermal test has a wheal diameter of at least 5 mm.

##### Clinical indications

Skin tests for venom allergy are indicated to confirm the presence of venom sensitization in patients who have had systemic reactions to insect stings (or repeated severe large local reactions) and are candidates for venom immunotherapy. Testing is also useful to distinguish among different types of venom (bee, wasp, etc);

##### Age limitation

No age limitation.

##### Description of adverse/unintended reactions associated with the procedure

Adverse events are very rare with venom skin tests. Local itching and induration is a normal positive response, and may take hours to subside. Anaphylactic reaction to venom skin tests is extremely rare. Unintended consequences of venom skin tests can occur when the tests are performed in individuals who have no clear history of anaphylaxis to a sting. This is because venom skin tests can be positive in 15–20 % of adults, and in more than 30 % of those who have been stung in the previous few months. A positive test in such individuals creates the perception of risk even when the history might indicate low risk (<3 %) of anaphylaxis. This is the case in people who have large local reactions to stings, in those with only cutaneous systemic reactions to stings, and in patients who have completed a 5 year course of venom immunotherapy.

1. Type and spectrum of adverse reactions
  Local:
    Local adverse reactions are very uncommon but might cause delayed progressive swelling and induration of the test site, with itching and possibly pain.
  Systemic:
    Systemic allergic reactions to venom skin tests are rare, and near-fatal or fatal reactions are exceedingly rare.
2. Prevalence and risk associated with the procedure:
  Early studies of venom skin tests included small numbers of subjects. They were focused on diagnostic accuracy, and reported no significant adverse effects. The only large study reporting on the safety of venom skin tests was that of Lockey et al [20]. In that survey of 3236 patients, 64 (2 %) had a systemic reaction during venom skin tests, 13 (0.4 %) of which were severe. Thirteen of 64 adverse reactions (20 %) were possibly vasovagal, and six other subjects (9 %) demonstrated no symptoms of immediate-type hypersensitivity. Thus, 45 (1.4 %) of the 3236 subjects tested had a systemic reaction that was considered to be a reaction of hypersensitivity, of which eight reactions (0.25 %) were severe.
3. Risk factors for adverse/unintended reactions:
  The risk of severe anaphylaxis is always increased in patients with a history of asthma. The severity of the previous reaction to a sting is not a risk factor for anaphylactic reaction to skin tests. In contrast to skin tests with inhalant allergens, the risk of anaphylaxis is not increased when intradermal tests are performed without initial prick/puncture tests. In fact, there are 2 studies of “accelerated” venom skin tests that reported no increased risk of adverse reaction [21, 22]. Guidelines and Practice Parameters in Europe and the United States do not recommend any precautions for venom skin tests in patients who are taking beta-blockers of ACEI medications.

##### Institutional/organizational safety recommendations

Guidelines and Practice Parameters in Europe and in the United States do not express any concern about safety of venom skin tests, and do not recommend any specific precautions or safety measures [19, 18, 23].

##### WAO safety recommendations

1. Site:
  Both outpatient clinic and hospital setting
2. Personnel:
  Tests are performed by personnel who have undergone training and proficiency testing. A physician should be present.
3. Emergency equipment availability:
  Should be available on site (mandatory)
4. Emergency staff (ICU) availability:
  Not required
5. Pretreatment:
  Not applicable
6. Duration of the supervised follow up after procedure:
  20 min
7. Contra-indications:
  See contraindications for SPT in previous section (inhalants)
8. Other considerations:
  The results of venom skin tests must be reviewed and interpreted by an experienced specialist in allergology, in the context of the clinical history of the patient and the natural history of the condition. The pitfalls of diagnostic allergy testing have been well-described [24].

#### Skin testing with drugs

##### Definition and short technical description

Skin Prick- (SPT) and Intradermal Skin Tests (IDST) are the most useful modality for demonstrating an IgE-mediated mechanism underlying clinical symptoms [25], whereas epicutaneous patch testing (or SPT/IDST with delayed reading) is the logical first step in defining the relevant drug in delayed cell-mediated hypersensitivity to systemically administered drugs – and not only for contact dermatitis caused by topically applied drugs [26, 27]. However, depending on factors such as the clinical type of reaction, the drug suspected, the pathomechanism of the reaction, the availability of qualified test substances and the existence of a valid test protocol, an individual approach must be chosen for any specific situation, i.e. drug testing has to be performed in an individualized manner.

Generally it is advised to perform the tests 6 weeks to 6 months after the hypersensitivity reaction. SPT can be done with any soluble drug, for the IDST sterility is important. Patch tests can be performed with any form of commercial drugs. In general, for most of the drugs there is a lack of standardization of reagent concentrations. Only recently a guideline has been released listing all the published and recommended test concentrations for any drug reported [28].

##### Clinical indications

Indications for SPT and IDST with drugs are immediate reactions manifested as erythematous eruptions/flushing, urticaria and angioedema, anaphylaxis, conjunctivitis, rhinitis and bronchospasm/asthma. The most common use of patch testing with drugs are maculopapular exanthemas, acute generalized exanthematous pustulosis (AGEP), and fixed drug eruptions [29, 30]. Other clinical entities where patch tests are being used are delayed-appearing urticaria, photosensitivity, drug reactions with eosinophilia and systemic symptoms (DRESS/DIHS), Abacavir hypersensitivity syndrome, Stevens-Johnson syndrome and toxic epidermal necrolysis (TEN) [31].

##### Age limitation

Skin testing with drugs can be performed at any age, although the experience in children is limited.

##### Description and prevalence of adverse/unintended reactions associated with the procedure

Skin testing with drugs is a safe diagnostic approach, if performed according to the published guidelines [26, 28, 32, 33]. However tests may be associated with some risk of adverse local and also systemic reactions; a relapse of the previous reaction might be provoked with any skin test procedure.

1. Type and spectrum of adverse reactions:
  Local:
    SPT, IDST and patch tests may cause local irritation resembling (false-) positive reactions. Skin necrosis and scarring might result from testing with toxic substances such as chemotherapeutic agents or when using non-physiological concentrations.
  Systemic:
    Anaphylaxis after SPT with chymopapain, penicillin, tetanus toxoid, and other drugs, has been reported rarely, leading to the conclusion that SPT is a safe diagnostic procedure, although a theoretical and remote risk in principle remains [10].
  IDST, being more sensitive than SPT, is more likely to induce systemic reactions. Urticaria, and rarely anaphylaxis have been described almost exclusively with β-lactams [reviewed in 25].
  Systemic reactions associated with patch testing are extremely rare. In a retrospective study that evaluated 111 and 134 patients with a history of severe cutaneous adverse reactions to drugs no severe side effects induced by the tests were reported [27, 30].
  Flare-up reactions:
    Flare-up reactions might be mediated by IDST and patch tests. A patch test-induced exfoliative dermatitis was observed in a patient with an adverse reaction to carbamazepine [34]. A relapse of a pruritic rash occurred following a prick test with pristinamycin [35]. The relapse of an AGEP has been provoked by patch testing with acetaminophen (paracetamol) while these tests remained negative [36]. Thus, patch tests, SPT, and IDST can induce a systemic reaction even though their results were negative [26].
  Fatalities:
    The few fatalities associated with skin tests, reported from 1895 to 1980, were associated with biologic products that are no longer used such as horse serum-derived tetanus or diphtheria toxins or pneumococcal antiserum [reviewed in 29]. A recent literature review on systemic reactions from skin testing concluded that the occurrence of systemic reactions with inhalant allergens has diminished over the last 30 years, whereas fresh food, hymenoptera venom and antibiotic SPT still carry some risk [10].
  Risk factors for adverse reactions:
    In general, patients with history of previous anaphylactic reactions, uncontrolled asthma or high degree of reactivity, small children or pregnant women,, may be considered at higher risk (i.e., they may react more readily and/or more severely to the minute test amounts applied [25].

##### Institutional/organizational safety recommendations

Several guidelines for performing drug skin tests have been published [26, 28, 32, 33].

##### WAO safety recommendations

1. Site:
  SPT and IDST with drugs should be performed in hospital settings of specialized centers. Since adverse reactions to drug patch testing are rare and rather not severe, tests can be applied at outpatient clinics.
2. Personnel:
  Trained technician or nurse under supervision of a physician. Personnel has to be prepared, trained and equipped for serious events, especially anaphylactic reactions.
3. Emergency equipment availability:
  Should be available on site (mandatory) for SPT and IDST. Not applicable for patch testing.
4. Emergency staff (ICU) availability:
  Should be available on site (mandatory) – only for patients who are tested for anaphylactic reactions, or for patients with a history of complicating conditions such as asthma, mastocytosis and severe cardiac disease.
  For all other patients should be available within 30 min
5. Pretreatment:
  Not applicable
6. Duration of the supervised follow up for safety after the procedure:
  Should remain in the centre for at least 20 min after completion of the procedure. After IDST in patients with previously diagnosed asthma (due to the suspected drug itself or as an underlying disease) supervised follow-up should be extended to 6 to 8 h [25].
7. Contraindications:
  Drug-induced autoimmune diseases, severe exfoliative skin reactions and severe vasculitis syndromes for SPT and IDST [25]. There are no absolute contraindications for patch testing with drugs.
  See also contraindications for SPT in previous section (inhalants)

#### Skin testing with occupational allergens

##### Definition and short technical description

The techniques for skin test (ST) with occupational allergens are identical to ST with other (inhalant) allergens. As for other allergens, in routine the skin prick test (SPT) should be preferred over the intradermal skin test (IDST), because it causes less pain and there is a lower risk of systemic reactions. Since there is no clear definition of occupational allergens (also food and drugs are occupational allergens for some workers) for the purpose of this document we looked for potential adverse reactions after skin testing in occupational exposed workers. The following sensitizing substances most commonly cause occupational asthma and are used for skin testing: dust of cereal flours, enzymes, laboratory animals, farming (animals, cereals, hay, straw and storage mites), fish and seafood as well as low molecular substances such as isocyanates, platinum salts and acid anhydrides [37, 38]. Natural rubber latex (hereinafter referred to as latex) is discussed separately (see following section on skin testing with latex). Due to the fact that occupational allergies in comparison to sensitizations to ubiquitous allergens are rare, often no standardized SPT solutions are available. In these cases non-standardized patient-tailored allergen preparations have to be used. If the patient shows a positive reaction to such a SPT solution, control tests should be performed in a number of healthy subjects in order to exclude an unspecific reaction.

##### Clinical indications

For the diagnosis of occupational type I allergies, the common steps are a detailed case history, skin testing, in vitro diagnosis (mostly specific IgE antibodies), and specific inhalation challenge. The clinical indication for SPT with occupational allergens (including latex) is to demonstrate IgE-mediated sensitization to occupational allergens. However, in combination with work-related symptoms of the patient, SPT with occupational allergens is also relevant for compensation and further socioeconomic consequences.

##### Age limitation

In general, STs with occupational allergens are performed only in working adults.

##### Description and prevalence of adverse/unintended reactions associated with the procedure

Taking into account, that skin tests with occupational allergens are only performed in adults and that commercial ST solutions for occupational allergens (other than latex) usually contain only small amounts of antigens and proteins [39] ST and especially SPT with occupational allergens remains a safe diagnostic procedure.

1. Type and spectrum of adverse reactions
  Local:
    If non-standardized patient-tailored allergen preparations are used, local adverse reactions might be caused by irritation or toxic reaction
  Systemic:
    In general the risk of systemic reaction following ST with occupational allergens is low (lower with SPT than IDST). There exists one report about an anaphylactoid reaction (without cardiovascular symptoms) after a scratch test with iridium chloride in an occupational exposed process operator [40]. However, scratch tests have generally been abandoned because of non-standardized procedure.
  Fatal:
    Out of 17 cases of anaphylaxis after SPT with various allergens listed by Liccardi [10] after Medline research (1980-2005) none referred to occupational exposure.
2. Risk factors for adverse reactions:
  Unknown

##### Institutional /organizational safety recommendations

EAACI position paper: skin prick testing in the diagnosis of occupational type I allergies [41]

##### WAO safety recommendations

Although allergy ST is considered a safe procedure, it is not without risk of systemic reaction

1. Site:
  Both outpatient clinic and hospital setting
2. Personnel:
  Can be performed by trained nurse/technician under supervision of experienced physician
3. Emergency equipment availability:
  Should be available on site (mandatory)
4. Emergency staff (ICU) availability:
  Not required
5. Pretreatment:
  Not applicable
6. Duration of the supervised follow up after procedure:
  Should remain in the centre for at least 20 min after completion of the procedure
7. Contra-indications:
  Identical to ST with other (inhalant) allergens
8. Other considerations:
  None

#### Skin testing with latex

##### Definition and short technical description

Natural rubber latex (NRL), commonly referred to as latex, is a vital natural resource that is used in the manufacturing of a wide variety of commercial products ranging from airplane tires to protective medical gloves. Ninety-nine percent of latex comes from one source: the sap-like fluid from the rubber tree *Hevea brasiliensis*. Sensitization to latex, which is a potent allergen, affects people who are frequently exposed to products made of latex such as health care and latex industry workers, patients with a history of multiple surgical procedures including children with spina bifida as well as specific food allergy patients.

Fourteen latex allergens have been identified and skin test (ST) extracts have to contain especially NRL allergens Hev b 1, 2, 3, 4, 6.01, 7.01, and 1 and recombinant Hev b 5 (rHev b 5). Skin prick test (SPT) should be performed, intradermal tests are not recommended (Cabañes et al. 2012) [42]. SPT extracts to determine latex allergy included commercial extracts, latex glove extracts and hevea leaves. Serial 10-fold dilutions of non-ammoniated latex (NAL, e.g. from Malaysian *Hevea brasiliensis* (clone 600) sap (Greer Laboratories)) or newly introduced ammoniated latex (AL, e.g. Bencard Laboratories, Mississauga, Ontario) allergens were employed in ST. Standardized extracts can provide a sensitivity of 93 % with a specificity of 100 % [42].

Also ‘glove use tests’ are performed. Considerable disparity exists between glove use protocols, with exposure times ranging from 15 min to 2 h. In general, the first step involves placing a fingertip of the glove on a dampened finger; if the result is negative, the complete powdered glove is put on. A vinyl or nitrile glove is used on the other hand as a negative control. The result is considered positive if contact causes erythema, pruritus, blisters, or respiratory symptoms [42].

##### Clinical indications

Latex is a common component of many medical supplies used in the hospital environment. Although latex is most often associated with disposable gloves, other items which may contain latex are breathing tubes, infusion sets, syringes, stethoscopes, catheters, dressings and bandages. Frequent users of latex products may develop a latex allergy. Allergic rhinitis and asthma mainly affect individuals exposed via inhalation, such as health care workers, lab workers, dentists, nurses, and physicians.

Patients at risk are also subjects with spina bifida and congenital genitourinary abnormalities who have undergone multiple procedures. While the incidence of latex allergy in the general population is 1 % to 2 %, in spina bifida (SB) patients, who are mostly children, incidence of latex allergy ranges from 20 % to 70 % [43]. As well, people who have certain food allergies, including banana, avocado, chestnut, apricot, kiwi, papaya, passion fruit, pineapple, peach, nectarine, plum, cherry, melon, fig, grape, potato, tomato and celery, may also have signs of a latex allergy due to cross-reactivity.

Diagnosis of latex allergy is based on clinical suspicion. A good clinical history taken by an experienced allergologist is very important. The history should record the presence or absence of other allergies, atopy, previous operations or medical procedures involving latex products, reactions induced by ingestion of fruits and whether the patient belongs to a risk group. The complementary diagnosis is based on STs and the determination of specific IgE [42].

##### Age limitation

SPTs with latex are performed both in adults and children.

##### Description and prevalence of adverse reactions associated with the procedure

Skin testing with latex allergen is associated with a significant risk of adverse systemic reactions

1. Type and spectrum of adverse reactions
  In most cases, subjects with adverse reactions after latex SPT showed a variety of different symptoms.
  Local:
    There are no reports about isolated large local reactions after latex SPT. One health care worker in whom angioedema, hives and hypotension developed had no discernible wheal and flare reaction at the site of the SPT (Kelly et al. 1993) [44].
  Systemic:
    Several reports of anaphylaxis during SPT for latex allergy have been published (Table 3). However, in these former cases mostly non-standardized SPT extracts prepared from powdered latex gloves or crude latex preparations directly from *Hevea brasiliensis* trees were used. In a study initiated with the goal to establish an FDA (Food and Drug Administration) – licensed extract for use in the United States an optimal diagnostic accuracy (SPT [IDST]: sensitivity 96 % [93 %], specificity 100 % [96 %]) without any systemic or large local reactions was obtained in 59 latex allergic adults with a non-ammoniated latex extract (SPT 100 μg/ml, IDST 1 μg/ml) [44]. More recently, the available standardized latex SPT reagents in Canada and Europe enable SPT with latex with a low risk of inducing systemic allergic reactions [45, 46]. However, so far there is no approved SPT solution for latex in the United States [47]. Not only SPT with latex per se is mentioned to be a putative risk factor for anaphylaxis during SPT but also a low age [9].

**Table 3:** Exemplary cases of systemic reactions during skin prick test (SPT) with latex

Number of cases reported	Type of ST	Culprit agent (type of latex)	Symptoms/fatalities	Reference	Comments (e.g. setting, age etc.)
1	SPT	Liquid latex material	Immediate flushing, tachycardia, urticaria, light-headedness	Spaner et al. 1989 [260]	34 year old female Operating room nurse
1	SPT	10 % aqueous dispersion	Cold, sweaty extremities, initial tachycardia, subsequent bradycardia, hypotension	Bonnekoh and Merk 1992 [261]	17 year old female dentist’s assistance
1	SPT	100 HEP *Hevea brasiliensis*	Dizziness, difficulty with breathing, wheezing, tachypnoea	Nicolaou and Johnston 2002 [262]	39 year old female house wife
6	SPT	extemporaneous extracts (dilutions of 1:1000, 1:100, and 1:10) (*n* = 5), commercial SPT solution (*n* = 1)	Signs of anaphylaxis in different degrees	Nettis et al. 2001 [48]	6 female patients with age ranging from 26-51
9	SPT	1:100,000 dilution of latex glove extract	Systemic reactions	Kelly et al. 1993 [44]	9 of 107 patients: 85 children with spina bifida, 15 health care workers, 7 others
2 or 3^a^	SPT	50 % glycerine, 0.23 mg/mL total protein from gloves	Pruritus, flushing, urticaria, angioedema, asthma, cough chest tightness, wheezing, dyspnea, eye itching, nasal congestion	Valyasevi et al. 1999 [15]	2 or 3^a^ patients from a clinic out of 1316

^a^in one patient reason of anaphylactic reaction was not clear because he was also positive to aeroallergens
  Fatal:
    The authors did not find reports that patients died after latex SPT. However, in cases of anaphylactic reactions emergency pharmacologic intervention was necessary [48] and patients have been hospitalized for continued therapy [44].
2. Prevalence and risk factors for adverse reactions
  Young patients with spina bifida may be at higher risk of systemic reactions with latex SPT. As a rule, patients with a positive bronchial challenge test result presented the most severe reactions [48]. It has been suggested that in patients with a history of latex allergy with systemic symptoms in-vitro tests should be performed before SPT [44].

##### Institutional/organizational safety recommendations

There are no published safety recommendations referring specifically to skin testing to latex allergens. The currently published position paper ‘Latex Allergy’ stated that standardized latex SPT extracts are considered safe, although isolated cases of anaphylaxis have been reported. Intradermal tests are not recommended [42]. The diagnostic algorithm for latex allergy which has been proposed by Hamilton et al. [46] may decrease the risk of adverse reactions during skin testing.

##### WAO safety recommendations

Skin testing with latex allergens in highly sensitive patients is considered to be associated with some risk of systemic reactions.

1. Site:
  Both hospital and outpatient setting
2. Personnel:
  Should only be conducted by allergy specialists or equivalently trained medical nurse/technician
3. Emergency equipment availability:
  Should be available on site (mandatory)
4. Emergency staff (ICU) availability:
  Should available within 30 min
5. Pretreatment:
  Not applicable
6. Duration of the supervised follow up after procedure:
  Time of supervised follow-up should be extended up to 40 min.
7. Contra-indications:
  Patients with a history of systemic reactions due to latex: no latex SPT or safety precautions essential
8. Other considerations:
  In patients with a history of latex allergy with systemic symptoms in-vitro tests should be performed before SPT. To reduce the likelihood of adverse reactions SPTs with latex allergen should be performed using different dilutions (beginning with higher dilutions).
  See also contraindications for SPT in previous section (inhalants)

### Bronchial provocation tests (BPTs)

#### Bronchial provocation tests with allergen

##### Definition and technical description

Inhalation or bronchial allergen challenge is a well-established and reproducible method to confirm sensitization to specific allergen in the bronchi. Allergen-induced reaction manifests as an early asthmatic response (EAR) and may or may not be followed by a more prolonged airway response (late asthmatic response; LAR).

Two methods can be used: continuous generation of an aerosol by a nebulizer and inhaled by the subject via a facemask or inhalation of a standardized dose of an aerosol by generating it intermittently. Any reproducible inhalation method can be used for either approach, the incremental allergen challenge usually employs 2-mins tidal breathing from a calibrated constant output nebulizer, while the single bolus method usually uses a counted number of deep breaths from a (breath-actuated, standard-dose) dosimeter [49, 50]. Both methods produce comparable airway responses.

The patient can also be challenged with allergen (e.g. wheat flour), with a use of a special/custom chamber, by the allergen as such especially in examinations for occupational asthma (e.g. baker’s asthma) [51] or in environmental exposure chambers for clinical research purposes. Bronchial response to allergen is either early, late or both and is verified by measuring lung volumes (FEV1) by flow-volume spirometry or peak expiratory flow (PEF) values. The patient is closely followed for at least 6 to 8 h after the challenge. In some centers segmental bronchial provocation techniques through fibro-optic bronchoscope have been employed.

##### Clinical indications

Allergen challenge is not a routine diagnostic procedure as patients are examined for their asthma or asthma suspicion and asthma diagnosis is obtained by other means. BPT with allergen is primarily a research tool in investigations on pathophysiology of asthma and on asthma controller therapy. However in some centers BPT with allergen is used to confirm sensitization and/or explain discrepancy between the clinical history, and the sensitization (skin tests and specific IgE).

Allergen provocation is also done to explore the causal relationship of allergen exposure at a workplace to patient’s actual symptoms (occupational asthma) [51]. For example, in the Finnish clinical routine these tests are rarely performed and only in occupational settings.

##### Age limitation

In clinical allergy practice inhalant allergen tests are seldom done to children or elderly, however no clear age limits have been established. Adults of working age are usually subjects of these tests, especially in occupational settings or research. There are centers where children are challenged with allergen (e.g. house dust mite) [52, 53].

##### Description of adverse/unintended reactions

1. Type and spectrum
  Local:
    Bronchoconstriction is developing from few minutes to 3 h and patient usually experiences some cough, chest tightness and even wheezing. These symptoms are usually easily to control with bronchodilators. From 16 to 50 % of the patients, dyspnea may appear after 3 to 8 h as a LAR [54].
    Severe asthma attack resulting in prolonged exacerbation of asthma sometimes occur. Other accompanying local symptoms may include irritation of throat, trachea and bronchi, causing cough.
  Systemic:
    Exceptionally, severe anaphylactic reactions caused by the allergen inhalation challenges can occur. Such reactions usually develop within few minutes and require epinephrine injection.
  Fatal reactions:
    One case of death caused by rapid, severe bronchoconstriction and anaphylaxis have been reported during exposure to isocyanate 30 years ago [53].
2. Prevalence
  No systematic review is available on the occurrence of unexpectedly strong bronchial responses or anaphylactic reactions. For occupational allergen inhalation challenge it is considered that 12 % required repeated administration of an inhaled short-acting bronchodilator, while few (3 %, 95 % CI: 1–5 %) induced an asthmatic reaction that required additional oral or IV corticosteroids [55].
3. Risk factors
  Unstable asthma (FEV1 below 70 %, recent hospitalization for asthma requiring oral corticosteroids).
  The risk of moderate or severe reaction was increased when the subjects were challenged with a LMW agent and when they were using treatment with an inhaled corticosteroid [55].

##### Institutional/organizational safety recommendation

Safety recommendations have been included in the state –of-the art documents published by the ERS/ATS, EAACI, or the French Society of Allergology [56].

##### WAO safety recommendations

1. Site:
  Hospital setting recommended. Outpatient clinic setting could be acceptable.
2. Personnel:
  The provocation test can be performed by a trained nurse/technician but only under the surveillance of a competent physician.
3. Emergency equipment availability:
  Should be available on site (mandatory)
4. Emergency staff (ICU) availability:
  Should be available within 30 min
5. Pretreatment:
  Is not necessary, but symptoms of positive reaction should be immediately relieved by the inhalation of short-acting b2-agonist or by nebulization (e.g. 2.5–5.0 mg of salbutamol). In case of more severe reactions oral or intravenous corticosteroids or epinephrine are administered.
6. Duration of the supervised follow up after procedure:
  The patient should be followed closely in a hospital setting for at least 7 h after provocation. The subject should never be left unattended during and following the challenge procedure and FEV1 should be closely monitored for at least 7 h post-challenge. After the last lung function measurement (usually at ≥ 7 h post-challenge), subjects should receive inhaled bronchodilators until the FEV1 returns within approx. 10 % from pre-allergen baseline. Only if this is achieved and the subject is clinically stable, subject can be sent home with the following precautions: secured transportation from research center to home address, provided with rescue medications, preferably not left alone at home, and emergency number(s) of on-call qualified physician who has been notified of the subject.
7. Contra-indications for BPT with allergen
  Uncontrolled asthma and /or FEV1 < 70 % of predicted
  Recent hospital admission or asthma exacerbation
  Spirometry-induced bronchoconstriction (i.e., less than 2 baseline FEV1 measurements out of 8 attempts within 15 %);
  Recent major surgery; severe disease of the heart, brain, digestive tract, liver, kidney
  Active, recent or chronic infections; immunological disorder; cancer, history of anaphylaxis
  Pregnancy
  Use of systemic beta-blockers
8. Other considerations:
  Allergen preparations employed in the challenge should be as standardized as possible. Furthermore, to prevent sensitization and/ or bronchoconstriction in sensitized investigators, an exhaust hood and/or (HEPA) filters should be used during allergen nebulization.
  Other GCP-based prerequisites relate to data quality and integrity, consist of:
  Adequate, well-ventilated challenge rooms with standardized humidity conditions within an irritant and smoke-free area,
  Regularly calibrated and serviced equipment meeting ATS/ ERS criteria,
  Standardized, validated SOPs,
  Qualified laboratory and pharmacy, complying to locally required standards.

#### Bronchial provocation with lysine-aspirin

##### Definition and short technical description

Bronchial challenge with a soluble form of aspirin (lysine – aspirin; L-ASA) is used to confirm a history of hypersensitivity reactions induced by aspirin or other NSAIDs in patients with an underlying chronic airway respiratory disease (asthma/rhinosinusitis/nasal polyps) and manifesting primarily as bronchial obstruction, dyspnea and nasal congestion/rhinorrhea [57]. Incremental concentrations of L-ASA are administered by a dosimeter-controlled jet-nebulizer in 30 min intervals and forced expiratory volume in 1 s is measured at 10, 20 and 30 min after each dose [58, 59]. The provocation is considered positive if at least 20 % fall in FEV1 as compared with post saline baseline value occurs [60]. Diagnostic inhalation challenges with other NSAIDs (indomethacin, sulpyrine, ketoprofen) have been also reported.

##### Clinical indications

An inhalation provocation test with lysine -aspirin is used to confirm hypersensitivity to aspirin or other NSAIDs in patients with cross-reactive, respiratory type of hypersensitivity. It is an alternative to oral aspirin challenge test which is the diagnostic gold standard, but brings some risks of systemic reaction (see section on Oral Drug Provocation Test). Inhalation test with L-ASA is faster to perform than the oral test, but it is less sensitive and negative result of an inhalation test does not exclude NSAIDs-hypersensitivity. The diagnostic value of L-ASA BPT has been documented only in patients with a history of respiratory type of hypersensitivity to ASA/NSAIDs - called Aspirin Exacerbated Respiratory Disease (AERD) or NSAIDs Exacerbated Respiratory Disease (NERD) - and is considered to be specific, reproducible, and generally safe method for NERD confirmation [61].

##### Age limitation

The test is usually performed in adults since NERD is rarely seen in children. However, a single study on L-ASA BPT challenges in children (aged 6–17 years) reported, similar to adults, general safety of this procedure [62]. Interestingly, in one child, urticarial symptoms were reported following L-ASA bronchial challenge.

##### Description of adverse/unintended reactions associated with the procedure

1. Type and spectrum of adverse reactions
  1. Local:
    Typical and expected symptoms include dyspnea and chest tightness accompanied with fall in FEV1 developing within 10–30 min after positive bronchial challenge. The symptoms can be easily relieved by inhaled/nebulized β2 agonist. In some patients an early prolonged reaction has been observed (fall in FEV1 developing with 2–3 h) [63], while in one study, several hours following lysine aspirin challenge the development of late bronchial symptoms was observed [64].
  2. Systemic:
    Bronchial reaction induced by inhalation of L-ASA may be accompanied by extrabronchial (nasal and/or cutaneous) symptoms in almost half of ASA-hypersensitive patients, and in some patients, inhalation of L-ASA results in development of isolated extrabronchial symptoms [65, 66]. Only a single case of severe, systemic reaction has been described in a patient with history of ibuprofen - induced dyspnea, but without typical asthma triad [67]. The reaction alter LysASA BPT started with facial flush, and generalized pruritus was followed by shortness of breath, cold sweating, and wheezing. Severe bronchoconstriction (75 % fall in FEV1) was associated with asphyxia and hypotonia. The patient fully recovered after administration of epinephrine, oxygen, a short acting, bronchodilator by inhaler, methylprednisolone, and volume expander.
  3. Fatal reaction:
    No fatal reaction has been reported
2. Prevalence and risk associated with the procedure:
  Although BPT with L-ASA is generally safe, it may be associated with systemic reaction, thus precautions during the procedure are necessary. The major risk is a significant bronchospasm, which however, can be easily relieved by appropriate treatment [68].
3. Risk factors for adverse reactions
  Low basal FEV1 (below 70 % of predicted), uncontrolled asthma, inappropriate increasing of the dose of inhaled Lys-aspirin.

##### Institutional/organizational safety recommendations

General safety recommendations has been presented by the HANNA/ENDA guideline [57]

##### WAO safety recommendations

The following WAO safety recommendations are proposed (Grade IV):

1. Site:
  4. Hospital or outpatient clinic setting
2. Personnel:
  Physician should be responsible for supervising the L-ASA bronchial challenge procedure, which may be performed by a nurse.
3. Emergency equipment availability:
  Should be available on site (mandatory)
4. Emergency staff (ICU) availability:
  Should be available within 30 min
5. Pretreatment:
  Is not necessary, but symptoms of positive reaction should be immediately relieved by the inhalation of short-acting b2-agonist or by nebulization (e.g. 2.5–5.0 mg of salbutamol). In case of more severe reactions oral or intravenous corticosteroids or epinephrine are administered.
6. Duration of the supervised follow up after procedure:
  Patient should remain under observation in the office/hospital for at least 1 h after the completion of an aspirin inhalation challenge. The FEV1 value should have returned to within 10 % of the prechallenge baseline, before discharge from the hospital. The patient should be provided with a peak expiratory flow (PEF) meter and record the PEF values before leaving the hospital and every 2–3 h until late evening. In the case of any respiratory symptoms and a 20 % decline in PEF value, the patient should take short acting β2-mimetic and contact the center.
7. Contra-indications:
  Uncontrolled asthma and/or FEV1 below 70 % of predicted
  A history of very severe anaphylactic reactions precipitated by aspirin or other NSAIDs
  Infection of respiratory tract within 4 weeks prior to the challenge
  Recent major surgery, severe disease of the heart, brain, digestive tract, liver, kidney
  Pregnancy
  Use of systemic beta-blockers
8. Other considerations
  Although associated with some risk of more severe reaction, Lys-ASA-BPT is generally considered safe, sensitive, specific and reliable diagnostics tool for confirming both AERD and NERD

#### Nonspecific bronchial provocations

##### Definitions and technical description

During NS-BPT, a patient inhales under laboratory conditions increasing doses (concentrations) of a potentially bronchospasm-inducing agent or is exposed to forced hyperventilation during exercise. After inhalation of each dose FEV1 is measured. A challenge is completed when a significant fall in FEV1 occurs or a maximal cumulative dose (concentration) is administrated. The stimuli used for non-specific BPT can be classified as direct (methacholine, histamine, leukotrienes or prostaglandins), and indirect (e.g. exercise, eucapnic voluntary hyperpnea, hypertonic saline, adenosine monophosphate, and mannitol) depending if they act directly on a specific airway smooth muscle receptor or release mediators from inflammatory cells [69–71].

##### Clinical indications

The test are used to confirm the presence or assess the degree of airway hyperresponsiveness, which is one of the main characteristics of asthma, and its measurement, using different methods, is important in establishing a correct diagnosis [72]. However, non-specific airway hyper responsiveness may be also present in other chronic respiratory conditions such as COPD, cystic fibrosis, or allergic rhinitis. These tests have been also used to monitor asthma treatment [73] and to monitor nonspecific bronchial hyperresponsiveness before and after bronchial challenges with specific occupational and non-occupational agents [74].

##### Age limitations

Since change in FEV1 is the primary outcome measure for these test***s***, the ability to perform reliable spirometric maneuvers is the major limitation. Therefore, the use of these testing methods is not recommended for those under the age of 6. There is no upper age limit to perform NS- BPT. The use of impulse oscilometry, instead of spirometry, may expand the age groups able to perform reliable spirometric maneuvers as it requires passive cooperation instead of active participation [75].

##### Description of unintended/adverse reactions associated with the procedure

1. Type and spectrum of adverse reactions
  Local:
    Cough is the most common side effect of these protocols [71]. Less common side effects include oropharyngeal pain and irritation, chest discomfort, and dizziness. There are isolated cases of angioedema and Vocal Cord Dysfunction reported in the literature. Clinical staff exposed to the bronchoprovocation agents are at increased risk of bronchospasm if they have asthma.
  Systemic:
    No systemic reactions except for cough or gag have been reported.
  Fatal:
    No fatal reactions following NS-BPT have been reported.
  The NS-BPT procedures are considered to be generally safe and adverse reactions are usually mild and fairly easy to control. Most patients recover spontaneously after the challenge test or after receiving a standard dose of a bronchodilator. Distressed patients respond very well to inhaled bronchodilators with or without oxygen supplementation.
2. Risk factors for adverse reactions/unintended reactions
  The risk of excessive reaction may be increased in individuals with low baseline lung function, if their asthma is not well controlled or during active respiratory infection.

##### Institutional/organizational safety recommendations

Both European and America guidelines propose safety measures and list contraindications for NS-BPTs [76–78].

##### WAO safety recommendations and contraindications

1. Site:
  Hospital or outpatient clinic setting – These procedures do not require hospital-based specialized centers and hospital admission is not n***e***cessary for the duration of the provocation [79].
2. Personnel:
  The procedure can be performed by trained technician/nurse who is familiar with the guidelines and knowledgeable about specific test procedures. Physicians who have expertise in the field should be readily available to manage acute asthmatic reactions or other complications.
3. Emergency equipment available:
  Should be available on site (recommended)
4. Emergency staff (ICU) available:
  Not required
5. Pre-treatment:
  No pre-treatment is necessary, however drugs which may potentially affect the reactions (see guidelines) should be withheld prior to the challenge [80].
6. Duration of supervised follow-up:
  Due to the characteristics of the agents used, late-phase reactions are not expected, except in rare cases after exercise tests. As a result, no special follow-up is needed after recovering from bronchospastic reactions.
7. Contraindications:
  Severe airflow limitation (FEV1, < 50 % predicted or < 1.0 L) (absolute contraindication)
  Moderate airflow limitation (FEV1 < 60 % predicted or <1.5 L(relative contraindication)
  Uncontrolled asthma
  Spirometry-induced bronchoconstriction (i.e., less than 2 baseline FEV1 measurements out of 8 attempts within 15 %);
  Recent major surgery; severe disease of the heart, brain, digestive tract, liver, kidney
  Active, recent or chronic infection
  Pregnancy
  Use of systemic beta-blockers
  Current use of cholinesterase inhibitor medication (for myasthenia gravis) for methacholine challenges
8. Additional for exercise testing:
  The European Respiratory Society suggested [80]:
  FEV1 greater than 75 % of the predicted normal value
  The patient with unstable cardiac ischemia or malignant arrhythmias should not be tested.
  Those with orthopedic limitation to exercise are unlikely to achieve exercise ventilation high enough to elicit airway narrowing.
  For patients over 60 years of age, a 12-lead electrocardiogram (ECG) obtained within the past year should be available.
9. Other considerations:
  Subjects should understand the procedure and be able to perform reliable spirometric maneuvers.

### Nasal provocation tests

#### Nasal allergen provocation tests

##### Definition and short technical description

Nasal allergen provocation test (NAPT) or nasal allergen challenge test is the method by which the nasal mucosa is challenged by instillation of allergen into the nasal cavities. NAPT assesses the nasal response to the suspected triggering allergen.

There are several methods by which NAPT is performed. Some clinicians perform it by spraying the allergen solution as aerosols into the nasal cavity, while others apply a small allergen coated paper disk on the inferior turbinate. Nebulization or instillation by pipette/dropper are other forms of NAPT. Yet another form of allergen challenge is by using special challenge chambers with controlled environments and precise delivery of agents [81]. Therefore, there is no standardized uniform method for performing NAPT, and so also for the precise criteria for evaluating the positive response, and grading for the risk of adverse events [82, 83].

##### Clinical indications

NAPT is performed to confirm the diagnosis of AR in the situation of discrepancy between the symptoms and the results of skin prick test (SPT) and/or serum specific immunoglobulin E (sIgE), to objectively assess disease severity and to monitor the response to pharmacologic treatment, for specific immunotherapy (SIT) in AR, to study the pathophysiological mechanisms of allergic inflammation, and to diagnose occupational rhinitis [84]. Nasal provocation tests are necessary for the diagnosis of local allergic rhinitis [85].

##### Age limitation

NAPT can be done in both adults and children. Upper age limit depends on the presence of contraindicating disease conditions.

##### Description and prevalence of excessive/adverse reactions associated with the procedure

1. Type and spectrum of excessive/adverse reactions
  Local:
    The chance of side effects is influenced by the concentration of allergen and by the method of allergen application. The appropriate dose of allergen for provocation can be estimated based on the dose of SPT. The dose of allergen that elicits a positive response (3 mm) of SPT can be used for NAPT. The starting dose can be 1:1,000 then increased by either factor of 3 or 10 [86, 87] NAPT performed by spraying allergen or applying a small disk on the inferior turbinate carries a lower risk as compared to the methods using nebulization or instillation of allergen solution by a pipette/dropper.
    Adverse reactions from NAPT can be divided into those upper airway reactions (mainly nasal) and lower airway reactions (bronchoconstriction). An excessive reaction of the upper airway due to NAPT is a severe nasal blockage or excessive nasal discharge. NAPT also carries a risk of a delayed reaction defined as the reappearance of nasal symptoms 3–12 h after NAPT [86, 88, 89]. Some researchers have reported that the immediate and late phase response of NAPT was 63 % and 37 %, respectively [90].
    Lower airway adverse reaction to NAPT (bronchoconstriction) can occur when the allergen enters directly into the lower respiratory tract via the larynx. The chance of allergens directly entering the lower airways also depends on the method of NAPT used.
  Systemic:
  No systemic reactions following NPTs have been reported
2. Risk factors for adverse reactions
  The possibility of excessive/adverse events of NAPT comes from either the use of excess allergen for NAPT or deposition of the allergen from nose/nasopharynx into the lower airways.

##### Institutional/organizational safety recommendations

Not available

##### WAO safety recommendations

1. Site:
  Outpatient clinic or hospital setting
2. Personnel:
  Technician/nurse with physician’s supervision
3. Emergency equipment availability:
  Emergency equipment should be available on site (mandatory)
4. Emergency staff (ICU) availability:
  Should be available within 30 min
5. Pretreatment:
  Not applicable
6. Duration of the supervised follow up after procedure:
  30 min
7. Contra-indications:
  Intense nasal obstruction or septal perforation
  Current nasal symptoms
  Within 4 weeks after viral or bacterial infection.
  Not well-controlled asthma, severe asthma or severe chronic obstructive pulmonary disease (COPD)
  Cardiopulmonary disease where epinephrine is contraindicated
8. Other considerations:
  Proper cooperation of the patient when performing NAPT (especially when performing by allergen nebulization or pipetting/dropping) is mandatory. The patient should hold one’s breath during allergen instillation to prevent the leaking of the allergen into the lower airways [91, 92].

#### Nasal aspirin provocation

##### Definition/description

Nasal challenge with lysine aspirin or ketorolac (United States) can be used to diagnose aspirin exacerbated respiratory disease (AERD). Baseline symptoms and measurements of the upper and lower airway are made and then incremental doses of the aspirin or NSAID are applied internally to the nose, as drops or spray with close monitoring of symptoms and airway measurements. The timing of response differs from allergen challenge- so 45 min is allowed between application and measurement. An increase in nasal symptoms (obstruction, rhinorrhea, sneezing, itching) plus an objective decrease in the upper airway of >25 % minimal cross sectional area or volume 0–12 cm on acoustic rhinometry is a positive response [60, 93].

If negative after a total of 150 mg lysine aspirin an oral challenge should be undertaken.

##### Clinical indications

Used to assess aspirin sensitivity in patients with rhinitis and/or polypoid rhinosinusitis, and/or asthma.

##### Age limitation

Usually done in adults since AERD is uncommon in children. Upper age limit depends on health status, particularly spirometry- see below. Avoid in pregnancy.

##### Description of adverse/unintended reactions associated with the procedure

1. Local:
  Since topical aspirin is applied to possibly sensitive tissue the reaction usually involves the upper airway first with predominant symptoms being nasal obstruction, rhinorrhoea, sometimes sneezing, itching. The lower airway may become involved, with asthma symptoms, but this is less frequent than with oral challenge and rarely severe. Laryngospasm reported following ketorolac challenge. About 5–10 % of subjects experience mild gastric irritation.
2. Systemic:
  Very rarely skin reactions such as urticaria and angioedema can occur, again less commonly than with oral challenge where some 25 % are affected.
3. Fatal:
  No fatalities have been reported using nasal aspirin challenge
4. Risk:
  Using nasal lysine aspirin the challenge dose can be very accurately controlled. 6 % of 131 subjects developed asthma symptoms, only 1.5 % showed a significant >20 % decrease in FEV1. Skin reactions occurred in 5.3 %, mainly urticaria, one patient developed facial angioedema [94]. Compared with the standard oral aspirin challenge and desensitization, intranasal ketorolac and modified aspirin challenge significantly attenuated the mean percentage decrease in FEV(1) values (8.5 % vs 13.4 %; *P* = .01) and decreased the percentage of extrapulmonary reactions (23 % vs 45 %; *P* = .002), particularly laryngospasm (7 % vs19%; *P* = .02) and gastrointestinal reactions (12 % vs 33 %; *P* = .001). This protocol was significantly shorter, lasting an average of 1.9 vs 2.6 days (*P* = <.001). In fact, 83 % of the patients completed the new protocol in less than 48 h compared with only 20 % in the oral challenge control group (*P* < .001) [93].
5. Risk factors for excessive reactions:
  Laryngospasm to aspirin-challenge inadvisable
  Failure to monitor nasal airway objectively
  Dose miscalculation

##### Institutional/organizational safety recommendations

EAACI/GA2LEN guideline: aspirin provocation tests for diagnosis of aspirin hypersensitivity [60].

##### WAO safety recommendations

1. Site:
  Both hospital and outpatient clinic setting
2. Personnel:
  Technician/nurse supervised by physician
3. Emergency equipment availability:
  Should be available on site (suggested).
4. Emergency staff (ICU) availability:
  Available within 30 min
5. Pretreatment:
  Pre-treatment with all usual asthma therapy on day of study permitted; nasal therapy stopped one week beforehand.
6. Duration of the supervised follow up after procedure:
  30 min
7. Contra-indications:
  History of anaphylactic to NSAIDs Other contraindications as listed in previous section (NPT)
8. Other considerations:
  Informed consent is mandatory.
  In very severe aspirin hypersensitivity, for safety reasons start with lowest dose.
  Use of acoustic rhinometry to monitor nasal airway is recommended (Nasal Inspiratory PeakFlow is less sensitive) [95].

### Nasal endoscopy

#### Definition and short technical description

Nasal endoscopy is the gold standard and the most valuable tool in the clinic to afford the diagnosis (presence, severity, etiology, and follow-up) of the majority of rhinologic pathologies [96–98].

Nasal endoscopy is performed by a flexible or rigid scope attached to a light source by glass fiber. For diagnostic examination, a scope with an optic angle from 0–45° is used with a caliber of 2,5–4 mm. Other optics (45–70°, 4 mm) are mostly used in surgery. Nasal endoscopy may eventually be preceded by local administration of anaesthetic and/or decongestive drugs. First, the bottom of the nose through the nasopharynx is to be inspected with an evaluation of the nasal septum, the lower turbinate and meatus, the choanae and the nasopharyx. Afterwards, the scope follows the edge of the middle turbinate towards the *rostrum sphenoidale*, with examination of the middle and upper turbinates, the mucus drainage from the sinuses, possible accessory ostia from the maxillary sinus, and the aperture of the sphenoidal sinus. At last, to get a view of the ostiomeatal complex, the ethmoidal bulla, the access to the frontal sinus, and the olfactory cleft must be attempted.

#### Clinical indications

The following reasons for nasal endoscopy can be considered in allergy practice:

- The physical examination of the nasal cavities and paranasal sinuses. The different structures of the nose can be evaluated: nasal septum, upper, middle and lower turbinates and meati, ostiomeatal complex, cavum, nasopharynx, and olfactory cleft. Even oropharynx and larynx can also be examined using flexible endoscopy. Although subjective in nature (physician interpretation) it can provide an objective evaluation of nasal signed (i.e. nasal congestion, rhinorrhea/postnasal drip). Despite being more difficult to perform, rigid nasal endoscopy usually provides a better examination view than flexible endoscopy and anterior rhinoscopy [99].
- To assess severity, and to follow-up (after medical or surgical treatment) of nasal and sinusal diseases [97, 98, 100, 101]:
- For differential diagnosis of sinus diseases e.g.
  - Structural abnormalities: septal deviation, turbinate hypertrophy, choanal atresia, or adenoid hypertrophy.
  - Nasal vasculitis, granulomatosis, or bleeding diseases.
  - Benign tumors and malignancies (unilateral versus bilateral)
- To obtain biopsies (i.e. nasal mucosa, nasal polyps, tumors) and microbiological samples for both disease diagnosis and translational research [99].

#### Age limitation (children, adults, elderly)

Nasal endoscopy is possible in all ages, including children [99, 102]. The only relative limitation is the lack of patient’s collaboration.

#### Description of adverse/unintended reactions associated with the procedure

1. Type and spectrum of adverse reactions:
  Nasal endoscopy adverse reactions are mainly local. When exploring the nasal cavities a very mild discomfort with sensation of foreign body, nasal itching, and sneezing is quite common. Contact of endoscope head with the nasal mucosa, mainly linked to some unexpected patient head sudden movement, can induce some burning or pain sensation and rarely minor epistaxis. Although possible, vasovagal reactions are very uncommon. When exploring the throat with flexible scopes, a nausea sensation can also be induced. There is no data in the literature reporting prevalence and risks associated with the nasal examination using nasal endoscopy.
2. Risk factors for unintended reactions:
  Non-compliant patients, mainly children, and patients with significant nasal septum deviations and/or risk of epistaxis constitute the relative risk factors for these adverse reaction.

#### Institutional/organizational safety recommendations

Both European (European Rhinologic Society [ERS], European Academy of Allergology and Clinical Immunology [EAACI]) and American (American Academy of Otolaryngology, Head and Neck Surgery [AAOHNS], American Academy of Allergy Asthma and Immunology [AAAAI]) scientific societies have produced a number of position papers which include the efficacy, safety, and main technical recommendations for the use of nasal endoscopy in daily practice, clinical trials, and sinonasal and skull base surgery [99, 103, 104].

#### WAO safety recommendations

1. Site:
  Both hospital and outpatient clinics
2. Personnel:
  Nasal endoscopic, either rigid or flexible, should be performed by a well-trained physician, either otorhinolaringologist or not (allergologist, pneumonologist, pediatrician, or even general practitioner).
3. Emergency equipment availability:
  Not required
  Comment: For potential minor epistaxis, a wet gauze or merocel pack as well as silver nitrate sticks for cauterization of potential minor nasal bleedings should be available. For unusual but potential vasovagal adverse reactions, a reclining chair or a litter should be available in the clinic.
4. Emergency staff (ICU) availability:
  Not required
5. Pretreatment:
  In general, no pretreatment is mandatory. If examination is difficult to perform, bothersome and/or painful, local anesthesia (lidocaine, cocaine) may be used [105]. A nasal decongestant may also be useful mainly in the presence of nasal deviation, turbinate hypertrophy or swollen mucosa. Both local nasal anesthesia and nasal decongestion may help the physician to have a better view of nasal cavities, turbinates, middle meatus, and nasopharynx as well as to make the patient feel more comfortable during the endoscopic examination.
6. Duration of the supervised follow-up after nasal endoscopy:
  After nasal endoscopy, patients do not need a special follow-up supervision. Only in the case of minor complications (nasal bleeding, vasovagal reaction) the patient may remain in observation in the clinic as needed (usually less than one hour).
7. Contraindications:
  There are no major contraindications for nasal endoscopy. Minor contraindications may be severe nasal hyperreactivity (can be solved by using local anesthesia), non-collaborative patients, and predisposition to nasal bleeding (nasal vasculitis or granulomatosis, Rendu-Osler syndrome).
8. Other considerations:
  The presence of nasal deviations, turbinate and adenoid hypertrophy and chronic rhinosinusitis with or without nasal polyps are usually considered as exclusion criteria in clinical trials investigating the effect of medications in allergic rhinitis. Since posterior nasal deviation and small size nasal polyps are not easily visualized using anterior rhinoscopy, a number of patients with this concomitant problems (10–15 % of general population has CRS and 4 % nasal polyps) may be wrongly included in such clinical trials.

### Food provocation tests

#### Definition and short technical description

The oral food challenge (OFC) test involves having a patient ingest a food gradually, in incrementally increasing doses, under medical supervision to determine if there is allergy or tolerance [105–109]. The food may be prepared and presented in the manner in which it is typically consumed, or its taste and texture may be masked by mixing it with other foods. When the food is presented in its natural form, the test is considered an “open” feeding. An open OFC is commonly used in clinical practice [110], but may introduce bias. The food is masked for single-blind or double-blind, placebo-controlled OFCs, the latter format being considered the least prone to patient and observer bias and is therefore considered the “gold standard” [107, 111]. Testing is performed when the patient is in good general health and without flares of atopic disease, has eliminated the food from their diet, is not using medications that interfere with interpretation of the test (for example antihistamines), or medications interfering with gastric digestion and threshold levels such as anti-ulcer drugs [112]. Patients are observed throughout the OFC, and the feeding proceeds unless the clinician diagnoses an allergic response. If a typical serving size of the food is ingested without symptoms, tolerance is diagnosed. This may require an open feeding of a larger amount following a masked feeding. If a reaction is elicited, treatments may be administered to reverse the allergic reaction, and the conclusion is that the patient is allergic. The time required for feeding the test substances (test food and placebo), and observing for reactions, varies depending upon specific protocol and the anticipated outcomes, e.g., immediate or delayed reactions, but usually takes several hours.

#### Clinical indications

An OFC is indicated to confirm that an allergic or other adverse reaction to a food exists [105–108, 113]. The test is recommended as a diagnostic procedure because, in contrast to a positive allergy skin or serum sIgE test that indicates sensitization but is not solely indicative of allergy, the OFC may verify or exclude clinical allergy [111, 114, 115]. An OFC is typically administered when other tests, including the medical history, skin testing and/or serum tests are inconclusive, and there is motivation to add the food to the diet or clarify the existence of the allergy [105]. Typical circumstances warranting an OFC include: suspicion of an allergy because of a possible allergic reaction, but having inconclusive supporting tests; no exposure to the food but having positive tests, or evaluating if an allergy has resolved when other tests remain inconclusive. The OFC test can detect immediate or delayed allergic and even non-immunologic reactions. An OFC may also be indicated for research purposes, or to determine an individual’s threshold of reactivity [107]. When making the decision to perform an OFC, the clinician should also consider the: risk of reaction (based on history and prior tests), potential severity of a reaction (may relate to the food tested, history, presence of asthma in the patient, test results), patient or family preferences, nutritional importance of the food, social aspects of being able to advance the diet, and emotional consequences should the food not be tolerated.

#### Age limitation (children, adults, elderly)

The test may be conducted at any age. The test can induce anaphylaxis; therefore, the physician should be confident in recognizing and treating anaphylaxis in the age group being tested, and understand the health issues that may increase adverse reaction risks or present contraindications in the age group tested (e.g., heart disease, obstructive lung disease, pregnancy, etc.) [107, 108].

#### Description of adverse/unintended reactions associated with the procedure

1. Type and spectrum of adverse reactions
  A physician-supervised OFC may induce allergic reactions that could range from mild to severe, including anaphylaxis. Food-induced anaphylaxis can be fatal [116]. Although severe reactions have been documented [117, 118], fatal reactions during supervised OFCs have not been reported. Nonetheless, fatal reactions can occur. Symptoms in the event of a reaction, which are expected and should be anticipated, most commonly affect the skin, followed by gastrointestinal and respiratory symptoms [118, 119]. Cardiovascular symptoms are uncommon but must be anticipated, especially in adults. Delayed and biphasic reactions are possible, but also uncommon [120].
2. Prevalence and risk associated with the procedure
  The likelihood of having a reaction and the severity of a reaction is not accurately predictable by current tests [105–107, 109, 121, 122]. Data regarding risk assessment is limited by biases introduced by patient selection and other factors (foods tested, age, clinical approach, definition of a positive test, etc.). Variability in patient selection likely accounts for the large range of OFC outcomes; for example studies in children report reaction rates of 19–48 % [111, 118, 120, 123]. The emotional impact, for example increasing anxiety, is also not predictable. Reactions are most often managed with antihistamines, but epinephrine, including more than one dose, may be needed [120]. Epinephrine is administered, in general, for under 10 % of challenges (overall challenges, including those without reactions) [111, 120, 124]. Additional treatments may be required (e.g., oxygen, intravenous fluids, bronchodilators, etc.).
3. Risk factors for adverse/unintended reactions
  It is presumed that risk factors for reactions include increasingly positive test results, the food tested, personal sensitivity and target organ reactivity (e.g., asthma) [105, 123]. Clinical decisions about stopping an OFC, for example continuing dosing if a reaction has not clearly occurred, but is suspected, may also introduce increased risk that must be weighed against benefits of confirming the allergy [105, 106]. Dosing amount, and frequency between doses, may play a role in outcomes, but this has not been systematically studied [105, 107, 125]. Reactions, including severe ones, may occur on the first dose of an OFC [119, 121]. Very slow and gradual dosing does not necessarily reduce the risk of allergic reaction severity [107, 125]. Using capsules to mask the food allergen is not generally recommended. The use of capsules may result in more severe or uncontrolled reactions because they may release allergen in an unpredictable fashion and also oral symptoms are bypassed [126].

#### Institutional/organizational safety recommendations

A number of safety recommendations have been promulgated by various organizations, expert panels and authors [105, 107, 113, 114].

#### WAO safety recommendations

1. Site:
  Both hospital and outpatient clinic setting
2. Personnel:
  Trained personnel, including a physician, with experience in the procedure and skill
3. Emergency equipment availability:
  Should be available on site (mandatory)
4. Emergency staff (ICU) availability:
  Should be available on site (in less than 5 min) or within 30 min depending on the risk assessment
5. Pretreatment (if any):
  Not applicable
6. Duration of the supervised follow up after procedure:
  Observation periods should be determined based on clinical circumstances, but generally a 1–2 h observation is suggested for patients who tolerate the full food dose during the OFC and at least 4 h when a significant reaction occurs; discharge instructions should include the possibility of late reactions (patients understand how to identify and treat);
7. Contra-indications:
  The test should not be performed when the food has recently caused a life-threatening reaction or if the patient has a chronic medical condition that would pose a health threat in the event of anaphylaxis (angina, cardiac disease, pregnancy, severe chronic lung disease, use of beta-blockers, etc.).
8. Other considerations:
  Recording a peak flow or spirometry may be considered and intravenous access should be secured if anaphylaxis or severe reactions such as enterocolitis [127] are likely, or if emergency intravenous access would be deemed difficult. Monitoring of the blood pressure may be necessary.
  It is important to select a starting dose that is likely below the patient’s reaction threshold. Upon positive food challenge the patient should be advised in regard to dietary restriction through trained personnel (e.g., dietician) and equipped with emergency medications (e.g., self-injectable epinephrine) for allergic reactions upon accidental exposure. Patients should be encouraged to eat the food on a regular basis after a negative test.

### Oral drug provocation test

#### Definition and short technical description

Oral drug provocation test (DPT) is the controlled administration of a drug to diagnose immune-mediated (allergy) and non-immune-mediated drug hypersensitivity reactions. Incremental doses of the drug are administered with the aim of inducing symptoms emulating those reported by the patient but at a very low scale and in a safe and controlled manner. DPT should be performed placebo-controlled, single blinded, and, in situations where psychological factors may be present, even double-blind. The rational approach is provided in different reviews [128–130] and protocols are published for several drugs but these have not been standardized [57, 61, 129, 131–135].

#### Clinical indications

DPT are usually performed if other less dangerous testing methods are not available or do not allow a firm conclusion, and the outcome is of clinical relevance to the patient. DPT may be carried out in the following situations:

- To confirm the presence of hypersensitivity in a patient with equivocal history
- To exclude hypersensitivity, when clinical history suggests it may not be the culprit drug, when the reaction does not appear to be drug hypersensitivity reaction, and when skin tests or in-vitro tests are not available.
- To assess cross-reactivity to related drugs (e.g. an alternative betalactam antibiotic or another COX-1 inhibitor in hypersensitivity to NSAID)
- To certify tolerance to an alternative drug

#### Age limitation

There is no data on the age limit for DPT. In general, the procedure is limited by the ability to objectively assess the supposed hypersensitivity reaction that may be elicited and the risk to the patient when the reaction is provoked.

#### Description of adverse/unintended reactions associated with the procedure

The intention of the DPT is to evoke a hypersensitivity reaction, however the magnitude of the reaction should be limited to the minimum. Thus the major task is to minimize the potential risk of development of generalized and/or severe reaction

1. Type and spectrum of adverse/unintended reactions
  Adverse reaction is anticipated to be similar in manifestations to those occurring in the hypersensitivity reaction, with a similar time kinetic but usually milder and of shorter duration. In practice it may vary from mild, local and transient to generalized and severe and in some instances potentially fatal.
2. Prevalence and risk associated with the procedure
  The prevalence and risk of adverse reaction is dependent on the correct assessment of causality of the hypersensitivity reaction, risk-benefit evaluation of the patient undergoing DPT, assessment of the general health of the patient on day of procedure, and compliance with the technical requirements of DPT [122, 124].
3. Risk factors for adverse/unintended reactions
  Patients with severe co-morbidities such as uncontrolled asthma, cardiac, hepatic, renal or other organ specific or systemic diseases which can be worsened or activated if the hypersensitivity reaction is provoked would be at higher risk. In such instances, DPT is considered only if the drug under suspicion is essential for the patient.

#### Institutional/organizational safety recommendations

The most comprehensive safety guideline on DPT is from the European Network for Drug Allergy (ENDA) [57]. Other guidelines are on the general aspect of the diagnosis and management of drug allergy [18, 62, 136–138].

#### WAO safety recommendations

##### General statement

All DPT must be preceded by an individual risk-benefit assessment [139].

1. Site:
  Both hospital and outpatient clinic setting
  DPT can be done in the clinic setting if previous reaction was mild [140]. Patients with more severe reactions should be hospitalized for DPT [128].
2. Personnel:
  DPT should only be carried out by a trained nurse/technician under the direct supervision of the allergist.
3. Emergency equipment availability
  Should be available on site (mandatory)
4. Emergency staff (ICU) availability
  Should be available on site (within 5 min) or within less than 30 min reach depending on the risk assessment.
5. Pretreatment
  There should not be any pre-treatment that may mask early signs of a reaction.
  H1-antihistamines should be discontinued (duration depending on the half-life of the preparation) before the procedure. Corticosteroids, anti-leukotrienes and tricyclic antidepressants may modify response to the challenge and should be reviewed. Medications that may cause problems if emergency treatment becomes essential e.g. ß-blocking agents have to be reviewed and decision made on whether to stop the drug prior to DPT [141].
6. Duration of the supervised follow up after procedure
  The duration of supervised follow-up after procedure is dependent on the expected time latency between drug ingestion and reaction onset based on the previous hypersensitivity episode. In general, immediate-type reactions need a short observation period, whereas delayed-type reactions in the history may necessitate similarly long observation periods after procedure. If a mild reaction has occurred during DPT, observation after stabilization is recommended for at least 2 h. After severe reactions, hospitalization is mandatory because of the possibility of biphasic episodes that can be lethal if not recognized early and treated adequately [142]. It is recommended that before going home, all patients are given an action plan that stresses when to seek medical attention and a number to call in case of emergency.
7. Contraindications:
  DPT should not be carried out when the hypersensitivity reaction is serious and potentially life-threatening including: anaphylaxis, drug hypersensitivity syndromes/drug reaction with eosinophilia and systemic symptoms, acute generalised exanthematous pustulosis, exfoliative dermatitis, erythema multiforme major/Stevens-Johnson Syndrome or toxic epidermal necrolysis, generalised bullous eruptions, vasculitis and other drug-induced autoimmune disease, or specific major organ involvement such as cytopenia. Pregnancy is considered a contraindication for DPT unless the drug is essential during pregnancy or delivery [128, 137, 143].
8. Other considerations:
  On the morning of the DPT, the patient should have had only a light breakfast and morning medications taken or omitted (as instructed by the attending allergist). The patient’s health status should be good, without any sign of allergy or viral infection. Blood pressure, pulse rate, peak flow meter reading (in asthmatics or when bronchospasm is anticipated), and intravenous cannula inserted (if the initial reaction was suggestive of a systemic reaction/anaphylaxis).
  Prior to each incremental provocation dose, blood pressure, pulse rate, peak flow meter reading (in asthmatics or if bronchospasm anticipated), and any new symptom/sign must be clearly recorded.

### Insect sting challenge

#### Definition and short technical description

Sting challenge (SC) is the ultimate standard for the diagnosis of insect venom allergy [144, 145]. During this procedure patient is deliberately stung by a living insect of the culprit species. Before SC the respective insect needs to be entomologically classified.

In general, a blinded, placebo-controlled procedure is not possible and incremental doses of culprit venom cannot be applied, making the SC test less controllable compared to other challenge tests in allergic patients. A thorough patient work-up and the evaluation of contraindications are, therefore, of eminent importance.

#### Clinical indications

Depending on the individual risk profile and culprit, insect venom immunotherapy (VIT) may not be effective in 5–20 % of the patients. SC aims to identify those individuals on maintenance VIT to assess effectiveness and who are not protected after 3–5 years of VIT. Although the standard management of insect venom hypersensitivity does not include SC in the United States [25, 146], the results of SC tests may help physicians to decide on whether VIT should be performed with a higher venom dose and are an invaluable research tool. If standard VIT is not effective (systemic allergic reaction at SC despite VIT), a higher maintenance venom dose will be used (usually 200 μg venom). Later on, VIT effectiveness may be re-evaluated by a subsequent, second SC (after the higher maintenance dose has been reached).

Additionally, results of SC may improve patient quality of life if it can be demonstrated that one does not develop an allergic reaction to a sting of the culprit insect (i.e., less anxiety about future sting reactions).

#### Age limitation

There is no age limit for SC. However, in patients who are not capable of understanding the procedure, and who cannot give their informed consent SC cannot be done.

#### Description of unintended/excessive reactions associated with the procedure

1. Type and spectrum of unintended/excessive reactions at sting challenge:
  Adverse reactions include pain at sting site, and local reactions which may be large. In case of VIT failure, systemic allergic reactions may occur varying between minor and very severe, and affecting respiratory and/or cardio-circulatory function.
2. Prevalence and risk associated with the procedure:
  Pain at sting site, and a minor local reaction (wheal, erythema, and swelling) is undesirable but is viewed as a normal sting reaction. Large local reactions with a diameter of more than 10 cm, and/or local reactions of a duration of up to several days are very rare. According to pooled data of observational or randomized studies systemic allergic reactions may occur in 18.0 % (range 0–59 %) of bee honeybee venom allergic patients and in 4.3 % (range 0–12.3 %) of yellow jacket (vespid) venom allergic patients [146]. The vast majority of systemic reactions are mild to moderate; however, cases of severe systemic allergic reactions and in the absence of an early efficient emergency therapy, even a fatal anaphylactic reaction have been described.
3. Risk factors for unintended/excessive reactions:
  Systemic reactions need to be accepted in order to identify treatment failure [147]. The general risk for the patient in terms of a life-threatening reaction is significantly lower in a medical setting when adequate treatment of symptoms is started immediately after first onset of symptoms, than at field sting. In patients on VIT, several factors determine the overall risk for a systemic allergic reaction at SC (thereby indicating VIT failure). Patients allergic to honeybee venom are at a higher risk for systemic allergic reactions than patients allergic to vespid venom [148–151]. Systemic allergic reactions during SC are also more likely in patients who are on ACE (angiotensin converting enzyme)-inhibitor therapy [53–150, 152, 153]. The rate of systemic allergic reactions at SC depends on the venom doses applied during VIT with higher therapeutic venom doses (individual or cumulative) decreasing this rate [148, 151, 154]. The risk-lowering effect of a higher therapeutic venom dose is not specific for the type of Hymenoptera venom used for VIT. Thus, during SC, the magnitude of risk reduction is the same irrespective whether the patient has received a double VIT (standard dose of two different venoms), or a double dose of the same venom [151]. Duration of VIT is inversely correlated with the risk for a systemic allergic reaction during SC [151]. Severe systemic allergic reactions which have been observed before SC during the build-up or maintenance phase of VIT are also associated with an increased risk for a systemic allergic reaction during SC [151, 154–156]. In addition, certain underlying diseases (mastocytosis) increase the risk for systemic reactions at SC [151, 157].
  Factors, which influence the severity grade of a sting reaction, have not been systematically investigated. However, mastocytosis is a clear risk factor for very severe sting reactions [157]. Finally, the severity of systemic allergic reaction will increase if the patient presents with severe co-morbidities such as asthma or cardiovascular diseases.

#### Institutional/organizational safety recommendations

Guidance on how to perform SC has been described, and a guideline was published by the Interest Group on Insect Venom Allergy of the EAACI [144, 158].

#### WAO safety recommendations

The patient must be screened for any contraindication to SC; risks, benefits and alternatives of the procedure shall be discussed with the patient, and written informed consent for the procedure must be obtained. If the patient is unstable (in case of an organ dysfunction), or if the patient requires a medication possibly disposing him to a higher risk, SC shall be postponed until conditions have been improved.

Drugs, which might ease symptoms of an allergic reaction (thereby evoking falsely negative results at SC) should be discontinued before SC. These drugs may include corticosteroids, H1-antihistamines, or anti-IgE antibodies. The respective half-life of the medication has to be considered when planning a SC. If an ACE-inhibitor therapy is indispensable it should not be discontinued just because a SC is planned. ß-blocking agents should be stopped prior to SC if possible.

Before SC, treatment protocols (indicating venom doses of at least 100 μg, and adherence to injection intervals) should be requested from patients who have been treated elsewhere. Patients should remain fasted for at least six hours before SC, and should not be on a medication potentially interfering with anaesthesia. Chronic diseases like asthma or arterial hypertension should be stable and the patient should not suffer from any relevant acute disease. Blood pressure, pulse rate, and, in asthmatics, pulmonary peak flow or FEV_1_ should be measured. Before SC, a peripheral intravenous cannula with a large bore should be inserted into all patients.

1. Site:
  Both hospital or outpatient clinic setting
  After SC, some patients will require a subsequent in-hospital surveillance or treatment. Therefore, SC should be performed at a site which is sufficiently close to a hospital specialized on emergency treatment.
2. Personnel:
  The insect can be put onto the patients’ skin and can be motivated to sting by a trained nurse or by other assistance personnel. SC shall be done under direct supervision of an allergist.
3. Emergency equipment availability:
  Should be available on site (mandatory)
4. Emergency staff (ICU) availability:
  Should be available within 30 min
5. Pretreatment:
  No specific pretreatment is necessary. Cardiovascular or bronchial diseases, which might represent a specific risk in the context of SC, should be treated to reach a stable situation.
6. Duration of the supervised follow up after procedure:
  Monitoring of all subjective or objective signs and symptoms is required during and after SC. After SC the patient should be monitored for at least two hours or longer, depending on the patient’s history and on the outcome of SC. After severe systemic allergic reactions, hospitalization is mandatory until complete recovery (minimum duration 24 h).
7. Contraindications:
  SC should not be done in patients who already had a systemic allergic reaction after a field sting while still being in the maintenance phase of VIT. In patients with repeated side effects during the maintenance phase of VIT, SC should not be performed unless a tolerance of VIT has been reached. Severe or poorly controlled cardiovascular/respiratory diseases (FEV_1_-value ≤ 70 %) as well as pregnancy are contraindications for SC.
  If medications which might lower the risk for systemic allergic reactions at SC cannot be safely withdrawn, they shall be continued. However, results of subsequent SCs must be interpreted with caution since there is an increased chance for falsely negative results, and the reaction to a later field sting may differ from that observed after SC.
8. Other considerations:
  For patients with mastocytosis and other risk factors an alleviated maintenance dose should be given from the start.

## Therapeutic procedures

### Subcutaneous Allergen Specific Immunotherapy (SCIT)

#### Definition and technical description of the procedure (SCIT)

Since the beginning of the twentieth century, allergenic vaccines were administered subcutaneously (SCIT). The favorable clinical results obtained in the early empirical attempts, rapidly lead to a widespread use of SCIT, which remained for decades the only form of allergen immunotherapy. Some clinicians occasionally attempted to use routes different from the subcutaneous one [159–161], but the alternatives to SCIT remained of very limited interest for many years [162].

SIT is started by increasing subcutaneous injections of allergen up to a maintenance dose. Several protocols of SCIT administration (mainly regarding the modality to achieve the maintenance dose) have subsequently been proposed and used in clinical practice, such as the “rush”, ultra-rush or the “cluster” [163, 164].

After reaching the maintenance dose, the interval between injections is usually increased to monthly, and continued for 3 to 5 years. For hymenoptera venom allergy, the interval between maintenance doses can be delayed to every 4 months [165], and performed life-long especially in patients with significant risk factors such as mastocytosis or previous severe sting reactions.

#### Indications to SCIT

Allergen-specific immunotherapy (SIT) is a “biological response modifier” that affects the immune response towards allergens at different levels. For this reason, SIT is currently considered a cornerstone of the management of allergic respiratory diseases (allergic rhinitis/asthma) and of Hymenoptera venom allergy.

#### Age limitation

Usually SCIT is considered in children 5 years and older.

#### Description of adverse/unintended reactions associated with SCIT

1. Type and spectrum of adverse events (AEs)
  After the earliest descriptions of AEs due to SCIT [165] these are generally classified according to a system introduced in Europe since the 1990s [166] (largely based on the Mueller’s classification for hymenoptera venom reactions) [167] and up-dated by the World Allergy Organization and other organizations [168, 169]. Nonetheless, other classifications have been repeatedly proposed (for review see 174), always distinguishing between local and systemic reactions, grading systemic reactions according to their severity and the number of organs involved, and distinguishing between early (<30 min) and delayed reactions [162, 163, 168, 170]. Local reactions are limited to the site of injection and include itching, swelling, pain or induration.
  Systemic reactions include rhinoconjunctivitis, bronchospasm, urticaria/angioedema, generalized itching, abdominal cramps sometimes ending as respiratory failure or shock. Other reactions are considered nonspecific (headache, tiredness, general malaise). Also vaso-vagal reactions due to the injection (nausea, vomiting, bradycardia, hypotension, sweating) may occur. This again proposes the distinction between “generic” systemic adverse events and overt anaphylaxis [171], since the definition of anaphylaxis itself has been repeatedly changed during the last years. In general the involvement of more than one organ/system strongly suggests anaphylaxis, as well as a clinically relevant drop in blood pressure, loss of consciousness or respiratory compromise [171, 172]. The more recent classification of systemic AEs, has been proposed by the World Allergy Organization [172]. The majority of the AEs are immediate (i.e. within 30 min) and therefore are presumed to be due to specific IgE, but delayed reactions may also occur.
2. Prevalence of adverse reactions and risk associated with procedure
  The fact that the injection of allergens in atopic subjects could cause AEs including severe reactions and even death, had been recognized and published since the early 1980s [173–175]. The rate of systemic reactions with SCIT largely depends on the administration schedule, the type of allergen and the survey method (e.g. controlled trial VS questionnaire-based surveys). When evaluating the data from literature, it has to be noticed that the practice of SCIT largely differs between USA and Europe. In USA, allergen mixtures are commonly used, and the extracts are at higher concentrations [176], whereas in Europe, the usual attitude is to vaccinate with few (1 to 3) allergens. On the other hand, the improved manufacturing procedures and quality of extracts, the improved standardization of allergen extracts, and the divulgation/education efforts [163, 164, 168], have probably contributed to the decline of severe/fatal reactions.
  The majority of data on the safety of SCIT come from the USA surveys that have been regularly conducted over the past 40 years. According to past and more recent surveys the occurrence of fatal adverse events is less than 1 per 2,500,000 injections [177–181], although the occurrence of fatal or near fatal events has progressively declined over the years [179, 182–184]. The occurrence of systemic AEs with SCIT is approximately 0.05–0.6 % of doses administered. On the other hand, no large population-based surveys have been conducted among the European Countries, and the data from clinical trials are largely incomplete [185]. To date, the largest safety survey on SCIT was conducted in Italy [186], which analyzed over 1,700 patients, showing a 3.3 % of systemic reaction rate with no fatalities.
3. Risk factors for adverse reactions
  Based on the available data derived from the large USA surveys as well as European data reports, severe and uncontrolled asthma seems to represent the most prominent risk factor for severe side effects [177–179]. Other factors indicated in the official position papers have to be considered as relative contraindications and should be considered individually [168]. This is especially true for children below the age of 5, where severe AEs are more difficult to recognize and to treat [168]. Another well recognized risk factor is human error, including wrong dosing administration, injection into vessels, and lack of emergency measures immediately available [187]. Although in the past it was reported that large local reactions are more frequently described in those patients who experience systemic reactions, it is now well accepted that on an individual basis large local reactions are poor predictors of future systemic reactions [188].
  Finally, it has been sometimes suggested that SIT can induce autoimmune diseases. Recently published data, however, demonstrate no increase in autoimmune disease, and thus recommendations state that it should not be considered a contraindication to the treatment [189]. Recent data have also shown no need to avoid its use in well controlled HIV infection [190, 191]. Pregnancy seems not to be a significant risk factor for SIT [191, 192]. Although a hypothetical risk can exist, based on pathophysiologic considerations, there is no evidence that the use of betablockers (especially the cardioselective ones) or angiotensin inhibitors enhance the risk of adverse events in patients taking SCIT [193, 194].

#### Institutional/organizational safety recommendations

EAACI, Immunotherapy Task Force. Standards for practical allergen-specific immunotherapy (2006 Alvarez-Cuesta E) [163]

Allergen immunotherapy: a practice parameter third update (2011 Cox L et al) [164]

Sublingual immunotherapy: World Allergy Organization position paper 2013 update [195]

#### WAO safety recommendations for SCIT

These WAO recommendations refer to subcutaneous form immunotherapy (SCIT) only. Sublingual immunotherapy (SLIT) has not been included in this document since the vaccine (drops or tablets) is NOT administered in a medically supervised setting. Although the first dose of allergen vaccine may be administered at the allergist’s office, it has never been a formal requirement.

1. Site:
  SCIT treatment may be started and continued in an outpatient setting
2. Personnel:
  Only trained allergist may initiate and supervise SCIT. The injections can be made by a nurse under physician supervision.
3. Emergency equipment availability:
  Should be available on site (mandatory)
4. Emergency staff (ICU) availability:
  Should be available on site (in less than 5 min) or within 30 min, depending on the risk assessment
5. Pretreatment:
  The use of premedication with oral antihistamines/oral antileukotrienes still remains a matter of debate. On one hand it has been claimed that premedication may delay or mask systemic reactions. On the other hand, it has been reported that premedication could reduce the frequency and severity of AEs. The strength of recommendation on this matter is still weak, thus its employment largely remains in the hands of the physician.
6. Precautions and duration of the supervised follow up after procedure:
  Although some of the adverse events of SCIT can be avoided, others occur unpredictably and without explanation. Immunotherapy should be administered with a 26- to 27-gauge syringe, and the injection should be given subcutaneously in the lateral/posterior portion of the arm. The skin should be pinched and lifted off of the muscles to avoid intramuscular or intravenous injection and the skin should be wiped with disinfectant before giving the injection.
  It is well known that some fundamental precautions can be taken to reduce the risk of severe/fatal AEs. First, the correct administration (i.e., patient’s name, batch, and allergen) has to be verified and recorded. As recommended in all guidelines it is essential that the patient have a careful examination and medical history taken [168–172, 174–187]. These include the objective assessment of current respiratory symptoms/signs (e.g. asthma/rhinitis), the evaluation of previous systemic reactions to SCIT (immediate or delayed), and the presence of any concomitant acute respiratory illness. When feasible, a Peak Expiratory Flow evaluation should be done, considering a value of less than 70 % of best predicted a warning signal [162, 163, 167]. After each injection the patient should be observed for at least 30 min.
7. Contraindications:
  Pregnancy has been always prudently suggested as a potential contraindication to SIT, and to SCIT in general, although no evidence was present in the literature. A recent survey demonstrated that the use of SIT in pregnancy, when clearly indicated, does not increase the risk of perinatal or foetal adverse events [191, 192]. The suggestion of not starting SCIT during pregnancy, and not stopping an already ongoing SIT remains valid, based on common sense.
8. Other safety considerations:
  Safety of maintenance of SCIT may be improved by monitoring of symptoms, appropriate adjustment of vaccine dosing etc.

### Venom immunotherapy

#### Definition and technical description of the procedure (VIT)

Venom immunotherapy (VIT) is so far the only effective treatment that prevents anaphylaxis and improves quality of life in patients with venom allergy [23, 196–198]. Several protocols of VIT have been described and used in clinical practice: Conventional protocols are started with weekly injections of increasing venom doses from 0.01 to 100 μg over 2 months. In rush protocols the increase to the maintenance dose is reached by daily increasing doses of venom for 2 to 3 days. In ultrarush protocols the increase to a total dose of 100 μg is reached by injections every 30 min in 3.5 h (Table 1) [196]. After reaching maintenance dose the interval of injections is increased from weekly to monthly, and 6–8 weeks from the second year. The recommended duration of VIT is 3 to 5 years, in patients with risk factors like mastocytosis or previous severe sting reactions, VIT may be continued indefinitely or as long as the risk of accidental stings remains. The maintenance dose of 100 μg protects over 95 % of wasp and ant venom allergic and 80–90 % of bee venom allergic patients from systemic allergic reaction (SAR) when re-stung [148, 196, 197]. In case of a SAR to a re-sting during VIT an increase of the maintenance dose to 200 μg protects most of these patients from further SAR [196].

#### Indications for VIT

US guidelines recommend VIT in all patients with a history of SAR and positive diagnostic tests – skin tests and/or venom specific serum IgE. Excepted are children with only cutaneous reactions [23]. European guidelines [196] do not recommend VIT also in adults with only cutaneous reactions, unless there are special risk factors or a severe reduction of QOL [198].

#### Age limitation

VIT may be given to children including those of pre-school age [23, 196], although the balance between discomforts versus benefits of treatment should be considered on an individual basis. In general, it is best to wait until a child is old enough to understand and accept the treatment. Elderly patients have an increased risk of very severe SAR to accidental stings with lasting morbidity, e.g. myocardial infarction, cerebral infarction or even fatal outcome [199, 200], which may be prevented by VIT. Although older age and comorbidities also increase the risk of reactions to VIT itself, these are usually milder and easier to manage than a field sting. Therefore, there is no upper age limit for VIT.

#### Adverse reactions associated with VIT

1. Type and spectrum of adverse reactions
  Local reactions at the injection site are common, and may be large (>5 cm in diameter) or last more than 24 h. Immediate-type adverse reactions are common during VIT. The majority of these are mild skin-only reactions, but some may be severe.
2. Prevalence of adverse reactions and risk associated with procedure
  The reported proportions of patients experiencing one or more significant reactions requiring medical intervention are 10–20 % for bee and *Myrmecia* ant VIT, and 5 % for Vespula VIT [23, 148]. Fatal reactions to VIT have not been reported. Rush and ultrarush protocols (Table 1) protect most patients more rapidly but may increase the number of SAR side effects [196, 201].
3. Risk factors for adverse reactions
  In addition to the species of venom used, risk factors for SAR due to VIT include older age, coexisting cardiovascular or pulmonary disease, antihypertensive medications, elevated baseline serum tryptase and mastocytosis [200–204]. Intercurrent illnesses (e.g. fever, infection) may also increase the risk of an adverse reaction. Dialysed aqueous venom and Aluminium hydroxide depot extracts have somewhat lower risks of SAR [205, 206].

#### Institutional/organizational safety recommendations

The most recent international guidelines addressing the issue of VIT safety are: The guidelines of the EAACI [195]. The practice parameter update 2011 of the AAAAI [23] and the WAO anaphylaxis guidelines 2013 [207].

#### WAO safety recommendations

1. Site:
  Both hospital and outpatient clinic settings
  VIT treatment may be started and continued either at a hospital or in the office
2. Personnel: technician/nurse/physician
  The treating physician should recommend and supervise VIT. The injections can be made by a nurse under physician supervision.
3. Emergency equipment availability
  Should be available on site (mandatory)
4. Emergency staff (ICU) availability
  Should be available on site (in less than 5 min) or within 30 min, depending on the risk assessment and the immunotherapy protocol used.
5. Pretreatment
  Pre-treatment with oral antihistamines during the dose build-up phase reduces the risk of SAR during VIT, and does not impact on the overall efficacy of VIT [208].
6. Precautions and duration of the supervised follow up after procedure
  xPrior to each VIT injection, the patient should be asked about: (i) reactions or unexpected symptoms following the last visit or injection, and; (ii) any new health problems including newly prescribed medications. Blood pressure and pulse rate should be routinely measured before every injection is given. Issues identified may lead to modifications as follows:
    Reactions on previous visits or injections consider reduced VIT dose
    Intercurrent illness consider delaying treatment
    Newly prescribed antihypertensive medications consider temporarily withholding antihypertensive medications for 24–48 h prior to each visit for VIT.
    Poorly controlled blood pressure or new onset (or worsening) of possible cardiac or lung disease (e.g. angina, asthma) consider pausing VIT until further investigations and/or stabilisation of condition.
  After each injection the patient should be observed for at least 30 min.
  If there is no SAR the next injection can be given or the patient can be discharged after 30 min of observation.
7. Contraindications
  Contraindications for VIT are concomitant active neoplastic and auto-immune diseases [194]. VIT should not be started during pregnancy but can be continued if well tolerated before pregnancy.
8. Other safety considerations
  Safety of maintenance VIT may be improved by careful monitoring of symptoms and appropriate adjustment of vaccine dosing.
  After an SAR the next injection dose should be decreased.
  Discharge may be considered 1 h after complete regression of all symptoms, but after a severe reaction (hypotension or hypoxemia), observation for a longer period should be considered.
  If there are repeated SAR due to VIT, pre-treatment with oral antihistamines on the evening before and 1 h before the following VIT injections should be considered [208].
  Other options are to switch to a conventional protocol using dialysed aqueous or Aluminium hydroxide depot preparations [205, 206], and pretreatment with Omalizumab [209].

### Oral Immunotherapy for Food Allergy (OIT)

#### Definition and short technical description

Oral immunotherapy is a promising concept for the treatment of food allergy. The majority of clinical trials focused on peanut, cow’s milk, and hen’s egg allergy [210, 211]. Meta-analysis revealed a substantially lower risk of reactions to the relevant food allergen in those receiving OIT [211]. There are several protocols for OIT used throughout the world [212]. In general, OIT starts with oral administration of very low doses of food protein, e.g. 2 mg of peanut protein [213], which is given on a daily basis. The doses are progressively increased over time. Regular dose increments, e.g. biweekly, are performed mostly under medical supervision [212]. When a defined target dose is reached, this maintenance dose, e.g. 800 mg peanut protein [213] is continuously administered on a daily basis and continued for several years. However, it is important to note that, in fact, maintenance dose ranged among various centers from 400 to 8000 mg of peanut protein [214]. Moreover, to date there is no recommended duration for OIT as long-term studies are still missing [211].

#### Clinical indications

OIT is a promising treatment approach, but it is associated with risk of adverse reactions, including anaphylaxis; it is therefore not currently recommended for routine clinical use [215]. Patients with peanut or tree nut allergy might especially benefit from OIT, as natural tolerance is rare. In addition, patients with persistent cow’s milk or hen’s egg allergy will be candidates. The objective of OIT is to achieve first a clinical desensitization, which means the tolerance to a certain amount of the allergen with an ongoing therapy, and later a long-term tolerance, which means the permanent loss of reactivity also after stopping OIT [212].

#### Age limitations

OIT could be performed at all ages; however, most OIT trials have been performed in children [210, 211].

#### Description of adverse reactions associated with the procedure

1. Type and spectrum of adverse reactions
  The most common adverse reactions are local, e.g., oral pruritus, or gastrointestinal, e.g., abdominal pain. More severe adverse reactions affect the respiratory tract, e.g., wheezing or multisystem reactions [210–212]. The development of allergic eosinophilic esophagitis has been described [216].
2. Prevalence of adverse reactions and risk associated with the procedure.
  Currently OIT is only recommended in controlled clinical studies until the short- and long-term safety profile is better known and understood [215].
3. Risk factor for adverse reactions
  Augmentation factors, e.g., infection, menses and exercise seem to be risk factors for adverse reactions.

#### Institutional / organizational safety recommendations

EAACI food allergy and anaphylaxis guidelines: diagnosis and management of food allergy [215].

#### WAO safety recommendations

1. Site:
  Both hospital and outpatient clinic settings; however, OIT is currently not recommended for routine clinical use [214, 215].
2. Personnel: technician / nurse / physicians:
  Physicians experienced with food allergy and specific immunotherapy should recommend and supervise OIT. During the build-up phase food should be administered by a nurse under physician’s supervision.
3. Emergency equipment availability:
  Should be available on site (mandatory)
4. Emergency staff (ICU) availability:
  Should be available on site (in less than 5 min) or within 30 min, depending on the risk assessment.
5. Pretreatment:
  Antihistamines or omalizumab can be considered
6. Precautions and duration of the supervised follow-up after procedure:
  During the build-up phase, doses are progressively increased over time, e.g. biweekly, under medical supervision [212, 213]. After each dose the patient should be observed for at least 2 h. The same dose is given at home daily until the next increase. In the maintenance phase the tolerated dose is given daily at home [212, 213].
7. Contraindications:
  Commonly, OIT is not performed in patients with unstable asthma or in pregnancy.
8. Other safety considerations:
  Doses should be adjusted during infection as this has been described as an important augmentation factor.

### Drug desensitization

#### Definition and short technical description

The term desensitization is used for procedures inducing clinical tolerance or tolerization to drugs eliciting hypersensitivity reactions [217, 218]. Rapid desensitization protocols are used for type I IgE/mast cell-mediated allergic reactions and slow desensitization protocols are used for type IV delayed drug hypersensitivity reactions [218] and other hypersensitivity reactions such as aspirin exacerbated respiratory disease (AERD) [138].

Desensitization procedures are based on protocols in which suboptimal doses of the drug allergens are re-introduced, starting at 1/100 to 1/1000 the target dose or lower for patients presenting severe reactions, and increasing at fixed time intervals by doubling or higher increments until reaching the target dose. These protocols introduce the sensitizing medication in few hours up to 6–8 h. Desensitization protocols allow allergic patients to receive their first line therapeutic agents to treat infections, cancer or chronic inflammatory diseases. The induction of clinical tolerance is temporary and largely depends on the half-life of the medication. The desensitization state persists from few hours, in the case of antibiotics administered every 6 to 8 h to several days in the case of aspirin. It is generally accepted that once more than two half-lives of the medication have spanned the patient is no longer desensitized and will need re-desensitization. Successful desensitization can be achieved in patients with IgE/mast cell mediated hypersensitivity reactions (allergy to beta lactams or other antibiotics) [219] and platinium salts [220, 221] who present symptoms including urticaria, angioedema, wheezing, laryngeal edema, nausea, vomiting, diarrhea or hypotension. Anaphylaxis in which tryptase levels are found elevated in serum is not a contraindication for rapid desensitization. Other hypersensitivity reactions (patients with aspirin exacerbated respiratory disease [222], non-beta lactam antibiotics [223], sulfonamides [224] and other chemotherapeutics including monoclonal antibodies [221]) have been successfully desensitized with different and slow protocols, some of them involving several days. Patients with chronic urticaria exacerbated by aspirin and other NSAIDs (named NSAIDs Exacerbated Cutaneous Disease-NECD) may be refractory to desensitizations [225] with few successful cases [226]. There are few standardized protocols for delayed reactions and caution has to be taken to avoid desensitization in patients with severe cutaneous or systemic reactions. Only patients with non-severe delayed reactions are candidates for slow desensitizations [218].

#### Clinical indications for drug desensitization

Rapid and slow drug desensitizations are indicated:

1. If the drug is considered first line therapy (e.g. patients with platin-sensitive recurrent ovarian cancer, cystic fibrosis patients with antibiotics allergy, patients with NSAIDS intolerance in need of dual antiplatelet therapy).
2. If the drug is more effective than the alternatives.
3. If non-cross reacting therapeutic agents are unavailable.
4. The drug administrated after desensitization has a unique therapeutic effect (aspirin in patients with NSAIDs – exacerbated respiratory disease complicated with nasal polyps).

#### Age limitation

Most published protocols assess clinical efficacy of desensitization in adult populations. There are several published desensitization protocols for children (desensitization protocols to antibiotics [227] and chemotherapy [228]). The success rate of adult and pediatric desensitization protocols is similar with a range from 50 to 100 %.

#### Adverse/unintended reactions associated with the drug desensitization

In 30 to 50 % of all desensitization procedures mild symptoms occur and there are no reported deaths resulting from a desensitization protocol. Anti-histamines are used commonly as pre-medications and some protocols are modified once patients have presented reactions to subsequent desensitizations [228].

1. Type and spectrum of adverse reactions
  Most of the desensitization protocols are well tolerated by the majority of patients. However, reintroduction of a drug to an allergic patient carries high risk including anaphylaxis.
  Reactions during rapid desensitization protocols can occur in minutes and can range from flushing and urticaria to hypotension and oxygen desaturation.
  During aspirin desensitization in patients with Aspirin Exacerbated Respiratory Disease (AERD) the tolerant state is achieved by repeating the provoking dose of aspirin so that aspirin sensitivity has to be demonstrated during the procedure. Thus, except for so called “silent desensitization” adverse reaction (respiratory or cutaneous) are intentionally evoked during the procedure, but the magnitude is controlled and limited by rapid administration of reliever drugs. These reactions can appear at every step of desensitization protocols. In patients desensitized to aspirin, breakthrough reactions usually occur after the oral dose of 45–60 mg of aspirin [229] but it can be seen at higher doses. Protocols for rapid desensitization to aspirin in patients with cutaneous symptoms differ from the AERD protocols [225, 230] in which the prevalence of adverse reactions is only up to 19 %.
  During intravenous rapid desensitization protocols most of the adverse reactions are seen when the drug is infused at the maximal concentration and during the last step of the protocol [221].
2. Prevalence and risk associated with the procedure:
  Side effects may complicate 12 to 52 % of desensitizations to antibiotics and from 4–33 % of desensitizations to chemotherapeutics and 29 % desensitizations to monoclonal antibodies [220, 221]. In repeated desensitizations the rate of adverse reactions decreases to less than 10 % with over 6–10 desensitizations [221] and the spectrum of reactions ranges from cutaneous reactions [228] to anaphylactic shock [220].
3. Risk factors for adverse/unintended reactions
  The severity of the initial hypersensitivity reaction is the most important risk factor, but other factors such as the time course of the HS reaction (in patients with delayed HSR it is most reasonable to hospitalize patients for longer time), the concomitant use of other medications such as beta-blockers and ACE inhibitors and the severity of the underlying disease need to be taken into consideration.
  For patients desensitized to chemotherapy and monoclonal antibodies the presence of atopy, a previous severe reaction and the presence of severe cardiovascular disease are risk factor for severe reactions. In addition patients on beta blockers and on ACE inhibitors are at risk for severe hypotension and cardiovascular collapse during desensitization.
  Risk factors for severe or moderate reaction during aspirin desensitization include: age: 30–40, duration of AERD less 10 years, FEV1 < 80 %, uncontrolled asthma, previous asthma –related ED visits and lack of antileukotriene pretreatment [229].

#### Institutional/organizational safety recommendations

Not available

#### WAO safety recommendations

1. Site:
  Both hospital and outpatient clinic settings
  Comment:
    All high risk desensitizations should be done in the intensive care unit. High risk desensitizations are those performed on patients with initial grade 3 anaphylactic reactions associated with hypotension and/or oxygen desaturation, patients with severe/unstable cardiovascular diseases and/or on beta blockers and patients with FEV1 < 70 %.
    Once high risk patients have presented a successful desensitization in the intensive care unit repeated desensitizations can be done in the outpatient setting provided resuscitation medications including epinephrine, oxygen and intubation materials are available.
    Patients with hypersensitivity reactions involving the skin and/or two organs without changes in vital signs can be desensitized for the first time in the outpatient setting with trained staff and emergency equipment available on site (see below).
    In patients requiring repeated desensitizations (desensitizations to chemotherapy, monoclonal antibodies), after an initial successful desensitization, subsequent procedures can be performed in outpatient settings.
    For patients with delayed drug hypersensitivity slow desensitization protocols can also be done as outpatient procedures based on the severity of the initial reaction and the disease being treated (allopurinol desensitization for gout)
2. Personnel:
  Desensitization should be supervised by well-trained, experienced allergists and nurses. One on one nursing should be available for each desensitized patient and an allergist should be available on site at less than 3 min of the desensitization procedure.
3. Emergency equipment availability:
  Should be available on site (mandatory)
4. Emergency staff availability
  Should be available on the site
5. Pretreatment
  Pretreatment with systemic steroids is not recommended unless required by current guidelines for cancer treatment (dexamethasone for taxanes administration) [231]. Pretreatment with H1 and H2 antihistamines is recommended for rapid desensitization for chemotherapy, monoclonals and antibiotics but no controlled studies have been done comparing outcomes of desensitizations with and without pre-medications [221, 232]. Whitaker et al. [233] indicated that pretreatment with antihistamines alone or with glucocorticosteroids did not reduce the risk of reactions in desensitized patients. Leukotriene receptor blockade with montelukast and prostaglandin inhibition with aspirin have provided excellent protection against severe reactions in patients desensitized to chemotherapy and monoclonals [234, 235]. In early trials with paclitaxel and docetaxel approximately 30 % of patients presented acute infusion reaction and pretreatment with antihistamines and glucocorticosteroids and slower infusion rate reduced the rate of adverse reactions to 10 % [236]. Based on these results many current chemotherapy regimens include pretreatment with corticosteroids, antihistamines and proton-pump inhibitors.
  Although some authors [217] do not recommend pretreatment with antihistamines as it may mask early signs of hypersensitivity reaction [138], current studies in populations of desensitized patients without pre-medications are lacking and no recommendations can be made.
  Leukotriene receptor antagonists may alleviate symptoms of breakthrough reactions in aspirin hypersensitive patients [237] by shifting reaction from bronchial to naso-ocular symptoms and these pre-medications are strongly recommended at the present time for all patients desensitized to aspirin.
6. Precautions and duration of the supervised follow up after procedure
  The severity of the initial hypersensitivity reaction is the most important risk factor, but other factors such as the time course of the HS reaction (in patients with delayed HSR it is most reasonable to hospitalize patients for longer time), the concomitant use of other medications such as beta-blockers and ACE inhibitors and the severity of the underlying disease need to be taken into consideration.
  Patients should be in stable condition (FEV1 > 70 % in patients with asthma) before the start of the desensitization. In patients with cystic fibrosis the baseline FEV1 may be substantially lower and risk assessment should be done but low FEV1 is not considered a formal contraindication for desensitization.
  Duration of the supervision depends on the initial hypersensitivity reactions. In case of immediate reactions, rapid desensitized patients to chemotherapy, monoclonal and antibiotics are supervised for 30 min in the hospital for acute post desensitization reactions and then for the next 24–48 h for delayed post desensitization reactions.
7. Contra-indications
  The contra-indications to drug desensitization may be absolute or relative, when the risk /benefit evaluation is performed.
  Absolute contraindications
    Previous severe/life threatening cutaneous drug induced disease (SJS/TEN, DHS/DIHS/DRESS, AGEP)
    Cutaneous and systemic vasculitis
    Drug induced autoimmune disorders
    Drug induced organ involvement (hepatitis, nephritis, cytopenias, pneumonitis)
    Immune complex disorders (serum sickness disease)
  Relative contraindications
    Treatment with beta blockers and ACE inhibitors
    Unstable underlying disease (asthma, coronary heart disease)
  Maculo-papular rashes are not contraindications for desensitization and slow protocols are generally successful.
8. Other considerations:
  Treatment of chronic disease should be continued, but drugs that can influence the course of reaction like beta blockers and ACE inhibitors should be discontinued at least 24 h prior to desensitization to avoid prolonged and intractable hypotension during anaphylaxis induced by the desensitization procedure.

### Treatment with Anti-IgE and other biologicals

#### Definition and short technical description

Omalizumab is a humanized monoclonal antibody directed against human IgE. It prevents binding of soluble IgE to the IgE receptor. This is currently the only monoclonal antibody directed against IgE which is licensed.

#### Indication

Omalizumab is indicated for adults and adolescent and children (6 years of age and above) with moderate to severe persistent asthma, who have a positive skin test or in-vitro reactivity to a perennial aeroallergen and whose symptoms are inadequately controlled with inhaled corticosteroids [238–244]. Although new clinical data suggest that there are also other patient populations with asthma or other allergy or atopy related conditions, who would benefit from with Omalizumab, the clinical indication is currently limited to the above described patient group. More recently omalizumab has been approved for use in chronic idiopathic urticaria [245].

#### Age limitations

Anti-IgE is indicated for adults and adolescence (12 years of age and above).

#### Description of adverse reactions

1. Anaphylaxis

The frequency of anaphylaxis attributed to Omalizumab is estimated to be at least 0.2 % of patients, based on an estimated exposure of about 57,300 patients from June 2003 through December 2006. Anaphylaxis has occurred as early as after the first dose of Omalizumab, but also occurs beyond 1 year after beginning of regularly scheduled treatment [246]. Omalizumab has received a box warning label by the FDA [247–249].

2. Malignancy

Although malignant neoplasms were observed in 20 of 4,127 (0.5 %) Omalizumab-treated patients compared to 5 of 2,236 (0.2 %) control patients in clinical studies, the direct relationship between this treatment and the development of malignancies is completely unclear. The observed malignancies in Omalizumab-treated patients included a variety of different types, including breast, non-melanoma skin, prostate, melanoma, and others [238]. However, the impact of longer exposure to Omalizumab, or the use in patients at higher risk for malignancies, is not known and the application of omalizumab in patients with preexisting malignancies contradicted [239].

3. Eosinophilic conditions

In rare cases, patients with asthma on therapy with Omalizumab presented a serious systemic eosinophilia, sometimes presenting with clinical features of vasculitis, consistent with Churg-Strauss syndrome.

4. Fever, arthralgia, rush

Some patients have experienced a constellation of signs and symptoms including arthritis, arthralgia, rush, fever and lymphadenopathy with an onset 1 to 5 days after the first of subsequent injections.

5. Parasitic infection

It is not clear if treatment with omalizumab may be associated with increased morbidity attributable to parasitic infections [240].

6. Immunogenicity

Omalizumab does not seem to be associated with development of immunogenicity [243].

#### Institutional/organizational safety recommendations

Not available

#### WAO safety recommendations

1. Setting:

Both hospital and outpatient clinic setting

2. Personnel:

All personnel, supervising the patient during and after the injection, should be trained to handle anaphylactic reactions.

3. Emergency equipment availability

Should be available on site (mandatory)

4. Emergency staff availability

Should be available on site (in less than 5 min)

5. Pretreatment

No pretreatment

6. Precautions and duration of the supervised follow up after procedure

Following immediate reaction and intervention in the rare case that anaphylaxis occurs, the patient should be further followed up in an appropriate emergency setting.

Consider in-office waiting time – 2 h for the first injection of omalizumab and then 30 min after each subsequent dose. Patient should have an epinephrine autoinjector available [250].

7. Contraindications

The use of Omalizumab is contraindicated in the patients with a history of severe hypersensitivity reactions to Omalizumab or any of the preparation’s ingredients. Furthermore Omalizumab should not be used to treat acute bronchospasm or status asthmaticus.

### Treatment with products from human plasma

#### Definition and short technical description

Products made from human plasma are increasingly being used also in the field of allergy and asthma treatment. They include preparations of human immunoglobulins [251, 252], used for subcutaneous and intravenous administration. More recently, also other plasma components have been isolated and made available in commercial preparations, which can be used for various other conditions. A prominent example is the C1 esterase inhibitor [253]. C1 esterase inhibitor is manufactured from human plasma, purified by a combination of filtration and chromatographic procedures. Several precautions have been implemented to reduce the risk of viral transmission, since this factor, as well as immunoglobulin preparations in general, are being derived from a large pool of donors.

#### Indication

Indications for the use of human immunoglobulin preparations are the treatment of primary humoral immunodeficiencies, chronic immune thrombocytopenic purpura, and others [251, 252, 254, 255].

C1 esterase inhibitor is indicated for routine prophylaxis against angioedema attacks in patients with Hereditary Angioedema (HAE) [254].

#### Age limitations

Immunoglobulins are available for all ages, but some C1 inhibitor preparations have limited recommendations in children.

#### Description of adverse reactions

All adverse reactions are related to direct or indirect effects and mechanisms, known to occur by human immunoglobulins and plasma preparations [256]. These are particularly:

- Hypersensitivity reactions
- Renal dysfunction and renal failure
- Thrombotic events
- Hyperproteinemia, increased serum viscosity, and hyponatraemia
- Aseptic meningitis syndrome (AMS)
- Hemolysis
- Transfusion related acute lung injury
- Volume overload
- Transmissible infectious agents
- Interference with laboratory tests, due to the passively transferred antibodies in immunoglobulin preparations

The following risk factors have been identified for the development of thrombosis:

- Advanced age, prolonged immobilization, hypercoagulable conditions, history of venous or arterial thrombosis, use of estrogens, indwelling vascular catheters, hyperviscosity and cardiovascular risk factors.
- Patients predisposed to renal dysfunction, including those with any degree of pre-existing renal insufficiency, diabetes mellitus, age > 65, volume depletion, sepsis, paraproteinaemia, or patients receiving no nephrotoxic drugs.
- The only serious adverse reaction observed in clinical studies with C1 esterase inhibitor was cerebrovascular accident. The most common adverse reactions observed have been headache, nausea, rash and vomiting.

#### Institutional safety recommendations

Not available.

#### WAO safety recommendations

1. Site:
  Both hospital and outpatients clinic settings
2. Personnel:
  All personnel in direct contact to the patient must be experienced in handling hypersensitivity reactions.
3. Emergency equipment availability:
  Should be available on site (mandatory)
4. Emergency staff availability:
  Should be available on site (in less than 5 min)
5. Pretreatment:
  Pretreatment regimen including analgesics, antihistamines, and/or anti-inflammatory medications, steroids, hydration may be indicated in some patients to avoid or diminish common adverse effects
6. Precautions during the supervised follow up after procedure:
  Based on the individual risk of the patient (see above), special precautions have to be taken before administering these preparations. They may include, but are not limited to: Periodic monitoring of renal function and urine output, assessment of blood viscosity, analysis of signs and symptoms of hemolysis, and the presence of anti-neutrophil antibodies and anti-HLA antibodies in both, the product and patient serum. These should be obtained in case of an increased index of suspicion.
7. Contraindications:
  Immunoglobulin preparations are contraindicated in patients who have a history of anaphylactic or severe systemic hypersensitivity reactions to the administration of human immunoglobulin. Administration is also contraindicated in IgA-deficient patients with antibodies to IgA and a history of hypersensitivity. Anaphylaxis has been reported with the intravenous use of immunoglobulin preparations and is theoretically possible following subcutaneous administration.
  The C1 esterase inhibitor is contraindicated in patients who have manifested life-threatening immediate hypersensitivity reactions, including anaphylaxis to the product.
8. Other considerations:
  In case of hypersensitivity reactions stop infusion of injection immediately. Have epinephrine immediately available for treatment.

## Management of emergencies in allergy practice

All medical staff involved in either diagnostic or therapeutic allergy procedures should be trained in the recognition and management of allergic emergencies including anaphylaxis.

### Recommended equipment and medications

The following equipment is necessary at the allergy office performing diagnostic allergy procedures:

#### Equipment

1. Trolley for patient to lie flat if needed
2. Oxygen and suction equipment, including tubing, masks etc.
3. Airway management equipment according to skill level (basic essentials are oral and nasopharyngeal airways, bag-valve-mask for ventilation)
4. Intravenous access cannulae (20-16G) and giving sets; needles and syringes.
5. Manual blood pressure cuff
6. Nebulizer mask (for inhaled/nebulized epinephrine)
  Note: In a hospital setting it is recommended that there also be immediate access to ECG, pulse oximetry and non-invasive blood pressure monitoring equipment, advanced airway management devices for intubation and cricothyrotomy, and an intravenous infusion pump.

#### Drugs and fluids

1. Epinephrine (2 packs of 5 ampoules of 1 mg/1 ml)
2. Corticosteroid for intravenous injection
3. Antihistamines for oral or intravenous use
4. Two 1 L bags of normal saline
  Note: In a hospital setting, it is recommended that there also be immediate access to (a) smaller bags of saline for setting up an epinephrine infusion according to local hospital protocol and (b) a second line vasoconstrictor (metaraminol or vasopressin)

### Management of adverse reactions

Algorithms for management of allergic emergencies have been described by several state- of- the-art documents available [206, 207, 257–259]. Management of excessive or emerging adverse reaction should be prompt, but must be preceded by assessment of the situation and should involve careful clinical assessment of a patient. Any intervention should be tailored to the type and severity of symptoms and vital signs.

1. Local/Mild reactions
  Allergy skin testing or allergen injection during immunotherapy which are associated with development of local redness, edema and pain can be relieve by local application of cold compresses followed by oral antihistamine. For mild allergy symptoms, such as hay fever or hives, an oral antihistamine may be sufficient. For stuffy nose, decongestant can be given and for itchy, watery eyes, allergy eye drops may be sufficient.
  Difficulty breathing or wheezing related to e.g. inhaled allergen or oral food challenge should be assessed by measurement of respiratory function (spirometry) and could be relieved by inhalation of 2 puffs of albuterol or other beta2-agonist from an MDI
  If other symptoms like swollen lips, tongue, tightness in the throat, hoarseness or trouble speaking occur they should be consider as potential developing laryngeal edema and injection of adrenaline should be considered. Similarly, symptoms such as nausea, abdominal pain, vomiting, tachycardia, anxiety or dizziness may herald development of anaphylaxis and should be treated accordingly.
  The patient even with mild symptoms related to the procedure should be observed continuously and any worsening of symptoms should be assessed as potential signs of anaphylaxis.
2. Severe allergic reactions
  If criteria for a diagnosis of anaphylaxis are met (that is, involvement of two or more organ systems, or the onset of cardiovascular collapse/hypotension, an appropriate treatment protocol (Table 4) should be initiated. The reaction (although rarely) may not respond to a single intramuscular dose of epinephrine, thus the supervising doctor should be prepared to escalate treatment. Reactions limited to the skin may settle without treatment and/or be managed symptomatically with oral antihistamines. Parenteral antihistamines should generally be avoided as there is no proof of benefit and they may themselves trigger the onset of hypotension. The efficacy of steroids is unknown and so their use is not recommended as a routine.

**Table 4:** Standard protocol for anaphylaxis management

1. Initial steps
• Call for assistance • Give epinephrine 1:1000 at a dose of 0.01 mg/kg IM in the lateral thigh (maximum 0.5 mg) • Lie patient flat with legs elevated unless this causes increased respiratory distress, in which case the patient may prefer to sit up. However, return to supine position if there is any deterioration in conscious state • Airway management (according to skills and equipment) if required • Document a simple systolic BP by palpation (radial/ brachial pulse) and then deflate the cuff to just below systolic pressure as a tourniquet and gain IV access. If equipment is available, start physiological monitoring (ECG, oxygen saturations, 5 minutely noninvasive BP) and give oxygen if severe respiratory distress and/or hypotension. If the patient is hypotensive, also: b. Give IV normal saline bolus 20 mL/kg c. Gain additional wide bore IV access (14G or 16G in adults) and prepare to give additional fluid and/or adrenaline infusion if the patient does not respond to initial management For upper airway obstruction/stridor, also: d. Continuous nebulization of epinephrine (5 mL of 1mg/ml)
2. If there is inadequate response, an immediate life-threatening situation or deterioration
• Repeat IM epinephrine injection every 3–5 min as needed or start an IV epinephrine infusion as per hospital guidelines/protocol. Monitor BP closely. Nausea, vomiting, shaking, tachycardia or arrhythmias in the setting of normal or raised BP is likely to represent adrenaline toxicity rather than worsening anaphylaxis If the patient remains hypotensive, also: • Further N/saline fluid boluses (up to 50 mL/kg in total) may be required in the first 20 min • In the hospital setting, consider adding a selective vasoconstrictor (see Table 1). When indicated at any time, prepare to initiate cardiopulmonary resuscitation (CPR) including standard IV adrenaline dosing if the patient goes into cardiac arrest. Prolonged CPR is indicated because the arrest is usually sudden (no preceding hypoxia) and potentially reversible
3. Disposition
• Consider to use systemic corticosteroids to prevent potential late phase reaction Severe reactions should be monitored for a minimum 4 h after the last dose of adrenaline

## Summary and recommendations

Diagnosis of allergic disorders may require intentional exposure of patients to potentially allergenic or irritating substances and sometimes involves deliberate induction of allergic symptoms to offending compounds during provocation tests. Intentional application to a sensitized patient of potentially dangerous substances (allergy vaccines) is also a part of routine management of allergic diseases. Unwanted, excessive or even dangerous reactions associated with these procedures can be minimized or even avoided if the procedure is performed in appropriate manner and setting, medical personnel are aware of its potential risk and are prepared to appropriately handle the situation.

Following review of available literature the group of WAO allergy experts, representing various continents and areas of allergy expertise, reports on risk associated with diagnostic and therapeutic procedures in allergy practice. Based on known/expected risk and taking into account existing allergy guidelines/recommendations a set of safety requirements for performing allergy procedures have been proposed. The consensus on safety requirements for performing specific procedures recommends appropriate qualifications of personnel, optimal setting where the procedure should be performed, necessary availability of safety equipment, access to specialized emergency service and required time of medical supervision. The group proposes also general recommendations which should be followed in allergy practice, regardless of the type of diagnostic/therapeutic procedure.

The general recommendations include:

1. Procedures for the diagnosis and treatment of allergic diseases should be performed by medical personnel (physician/nurse/technician) fully aware of risks associated with the procedure and trained in the recognition and management of allergic emergencies, including anaphylaxis.
2. Some procedures can be performed by trained nurse/technician, but always under close supervision of the allergist.
3. Although most procedures can be done in both outpatient and hospital settings availability of appropriate rescue service should be secured.
4. Basic emergency equipment and rescue medications should be available on site during each allergy procedure.
5. Depending on the type of procedure emergency staff (ICU) should be available on site or should be reached within a specified time.
6. Before a procedure is initiated, contra-indications should be considered and risk/benefit ratio for each procedure should be assessed.
7. The patient should receive full information on the purpose and potential adverse effects associated with each procedure and for some procedures should be asked to sign an informed consent.
8. If anaphylaxis or severe reactions are likely, intravenous access should be secured before the procedure is started.
9. Continuous monitoring of patient by authorized personnel during is the procedure necessary to secure safety of performed procedure.
10. After the procedure is completed the patient should remain under close supervision for a specified period of time.
11. Before the patient is released, she or he should be provided with appropriate instruction in how to handle potential adverse symptoms and what to do in case of an emergency.
12. Medical personnel who have asthma or had a prior reaction to a testing agent should take precautions to minimize exposure (adequate ventilation, exhalation filters, hoods or closed chambers) or avoid performing these tests.
