# Erratum to: Risk and safety requirements for diagnostic and therapeutic procedures in allergology: World Allergy Organization Statement

**DOI:** 10.1186/s40413-016-0134-z

**Published:** 2017-01-11

**Authors:** Marek L. Kowalski, Ignacio Ansotegui, Werner Aberer, Mona Al-Ahmad, Mubeccel Akdis, Barbara K. Ballmer-Weber, Kirsten Beyer, Miguel Blanca, Simon Brown, Chaweewan Bunnag, Arnaldo Capriles Hulett, Mariana Castells, Hiok Hee Chng, Frederic De Blay, Motohiro Ebisawa, Stanley Fineman, David B. K. Golden, Tari Haahtela, Michael Kaliner, Connie Katelaris, Bee Wah Lee, Joanna Makowska, Ulrich Muller, Joaquim Mullol, John Oppenheimer, Hae-Sim Park, James Parkerson, Giovanni Passalacqua, Ruby Pawankar, Harald Renz, Franziska Rueff, Mario Sanchez-Borges, Joaquin Sastre, Glenis Scadding, Scott Sicherer, Pongsakorn Tantilipikorn, James Tracy, Vera van Kampen, Barbara Bohle, G. Walter Canonica, Luis Caraballo, Maximiliano Gomez, Komei Ito, Erika Jensen-Jarolim, Mark Larche, Giovanni Melioli, Lars K. Poulsen, Rudolf Valenta, Torsten Zuberbier

**Affiliations:** 1 Department of Immunology, Rheumatology & Allergy, Medical University of Lodz, 251 Pomorska Str, 92-213 Lodz, Poland; 2 Department of Allergy and Immunology, Hospital Quiron Bizkaia, Bilbao, Spain; 3 Department of Dermatology, Medical University of Graz, Graz, Austria; 4 Microbiology Department, Faculty of Medicine, Kuwait University, Kuwait City, Kuwait; 5 Swiss institute of Allergy & Asthma research, Davos, Switzerland; 6 Allergy Unit, Dermatology Clinic, University Hospital Zürich, University Zürich, Zürich, Switzerland; 7 Kirsten Beyer, Charité Universitätsmedizin Berlin, Klinik für Pädiatrie m.S. Pneumologie und Immunologie, Berlin, Germany; 8 Hospital Reg. Univ. Carlos Haya, Allergy Serv, Malaga, Spain; 9 Royal Perth Hospital, Department of Emergency Medicine, Perth, WA Australia; 10 Department of Otolaryngology, Siriraj Hospital, Mahidol University, Bangkok, Thailand; 11 Allergology Unit, Hospital San Juan de Dios, Caracas, Venezuela; 12 Brigham& Women’s Hospital, Harvard Medical School, Boston, MA USA; 13 Department of Rheumatology, Allergy & Immunology, Tan Tock Seng Hospital, Singapore, Singapore; 14 Hôpitaux Universitaires de Strasbourg, Chest Diseases Department, Strasbourg, France; 15 Department of Allergy, Clinical Research Center for Allergology and Rheumatology, Sagamihara National Hospital, Sagamihara, Kanagawa Japan; 16 Emory University School of Medicine, Atlanta Allergy & Asthma, Atlanta, Georgia; 17 Johns Hopkins University, Baltimore, MD USA; 18 Helsinki University Central Hospital, Helsinki, Finland; 19 Institute for Asthma & Allergy, Bethesda, MD USA; 20 Westmead Medical Centre, Westmead, NSW Australia; 21 Department of Paediatrics, Yong Loo Lin School of Medicine, National University of Singapore, Singapore, Singapore; 22 CSK, Department of Allergy & Clinical Immunology, Lodz, Poland; 23 Spitalnetz, Bern, Switzerland; 24 Rhinology Unit & Smell Clinic, ENT Department, Hospital Clínic, Clinical & Experimental Respiratory Immunoallergy, IDIBAPS, and CIBERES, Barcelona, Spain; 25 UMDNJ –Rutgers Medical School, c/o Pulmonary and Allergy Associates, Summit, New Jersey USA; 26 Department of Internal Medicine, Ajou University School of Medicine, Suwon, South Korea; 27 University of South Florida, Tampa, FL USA; 28 Allergy and Respiratory Diseases, IRCCS San Martino Hospital IST, University of Genoa, Genoa, Italy; 29 Department of Pediatrics, Nippon Medical School, Tokyo, Japan; 30 Universitatsklinikum GI & MR GmbH, Institut furLaboratoriumsmedizin & Path, Standort Marburg, Marburg, Germany; 31 Klinikum der Ludwig-Maximilians-Universitat, Klinik & Poliklinik furDermatologie & Allergologie, Munchen, Germany; 32 Allergy and Clinical Immunology Department, Centro Medico-Docente La Trinidadad, Caracas, Venezuela; 33 Allergy Department, Fundacion Jimenez Diaz, Universidad Autonoma de Madrid, CIBER de Enfermedades Respiratorias (CIBERES),Institute Carlos III, Madrid, Spain; 34 RNTNE Hospital, London, UK; 35 Division of Allergy and Immunology, Department of Pediatrics, Jaffe Food Allergy Institute, Icahn School of Medicine at Mount Sinai, New York, NY USA; 36 University of Nebraska, Omaha, NE USA; 37 Institute for Prevention and Occupational Medicine, German Social Accident Insurance, Ruhr-University Bochum (IPA), Bochum, Germany; 38 Division of Experimental Allergology, Department of Pathophysiology, Allergy Research Center of Pathophysiology, Infectiology & Immunology, Medical University of Vienna, Vienna, Austria; 39 Allergy & Respiratory Disease Clinic, DIMI –Department Int Med, University of Genoa, IRCCS AOU, San Martino–IST, Genoa, Italy; 40 Immunology Department, Universidad De Cartagena, Cartagena, Colombia; 41 Hospital San Bernardo, Salta, Argentina; 42 Department of Allergy, Aichi Children’s Health and Medical Center, Aichi, Japan; 43 Messerli Research Institute, Medical University Vienna, University Vienna, Vienna, Austria; 44 Department of Biochemistry and Biomedical Sciences, McMaster University, Hamilton, Canada; 45 Istituto Giannina Gaslini, Genoa, Italy; 46 Gentofte University Hospital, Lab for Allergology, Allergy Clinic, Hellerup, Denmark; 47 Department of Pathophysiology, AKH, Wien, Austria; 48 Campus Charite Mitte, Klinik fur Dermatologie & Allergologie, Berlin, Germany

## Erratum

Unfortunately, the original version of this article [1] contained an error. The spelling of author name Vera van Kempen was incorrect. The correct spelling can be found below.

Vera van Kampen
